# Liver-derived ketone bodies are necessary for food anticipation

**DOI:** 10.1038/ncomms10580

**Published:** 2016-02-03

**Authors:** Rohit Chavan, Céline Feillet, Sara S. Fonseca Costa, James E. Delorme, Takashi Okabe, Jürgen A. Ripperger, Urs Albrecht

**Affiliations:** 1 Department of Biology, Unit of Biochemistry, University of Fribourg, Fribourg 1700, Switzerland

## Abstract

The circadian system has endowed animals with the ability to anticipate recurring food availability at particular times of day. As daily food anticipation (FA) is independent of the suprachiasmatic nuclei, the central pacemaker of the circadian system, questions arise of where FA signals originate and what role components of the circadian clock might play. Here we show that liver-specific deletion of *Per2* in mice abolishes FA, an effect that is rescued by viral overexpression of *Per2* in the liver. RNA sequencing indicates that *Per2* regulates β-hydroxybutyrate (βOHB) production to induce FA leading to the conclusion that liver *Per2* is important for this process. Unexpectedly, we show that FA originates in the liver and not in the brain. However, manifestation of FA involves processing of the liver-derived βOHB signal in the brain, indicating that the food-entrainable oscillator is not located in a single tissue but is of systemic nature.

## Abstract (web summary)

Food anticipation is thought to be initiated by the central clock in the brain. Here the authors show that the peripheral organs initiate this process by showing that liver-specific deletion of Per2 can inhibit food anticipation by interfering with ß-hydroxybutyrate production and its subsequent processing in the brain.

## Graphical abstract

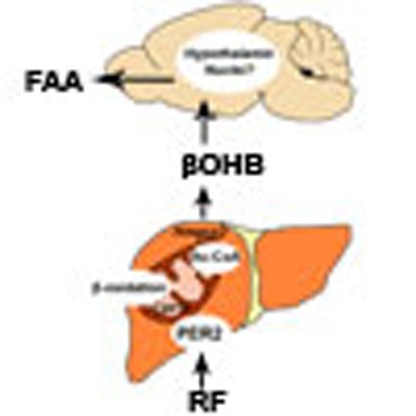

The circadian (∼24 h) timing system is a network of brain clocks and peripheral oscillators that enable mammals to adapt to daily recurring events such as light/dark (LD) changes and availability of food^1^. A population of coupled circadian clock cells in the hypothalamic suprachiasmatic nuclei (SCN) functions as master pacemakers responsible for the coordination of circadian oscillators in the brain and peripheral tissues to the daily LD cycle^2^. In many tissues, circadian clocks can also be entrained by daily cycles of food availability, which can uncouple circadian oscillators in peripheral tissues from the central pacemaker in the SCN^3^,^4^. While the SCN remains coupled to the LD cycle, peripheral oscillators such as the liver align with the daily feeding time. This is associated with the emergence of a daily bout of activity, rise in body temperature and increase in corticosteroids that precede meal time by 1–3 h (refs 5, 6). Remarkably, this so-called food anticipation (FA) exhibits the formal properties of a circadian clock, but persists robustly after removal of the SCN^7^,^8^.

At the molecular level, circadian rhythms are generated by a set of clock genes constituting an autoregulatory transcriptional/translational feedback loop^1^. The positive factors *Bmal1* and *Clock/Npas2* bind as heterodimers to E-boxes present in the promoters of target genes such as *Period* (*Per1, 2, 3*), *Cryptochrome* (*Cry1, 2*) and *Rev-erb* (*α and β*) to activate their transcription. PER and CRY proteins heterodimerize and enter the nucleus to inhibit the action of the BMAL1/CLOCK/NPAS2 activator complex, whereas REV-ERB inhibits transcription of *Bmal1/Clock/Npas2* genes, thereby closing the loop. The cycle is modulated by posttranslational events such as phosphorylation to modulate the speed of the cycle. Mice-lacking *Bmal1* (ref. 9), *Clock*^10^ and *Per1* (ref. 11) display normal FA, whereas in mice-lacking *Npas2* (ref. 12) and *Cry1/2* (ref. 13) FA is reduced. In contrast, deletion of part of the PAS domain in *Per2* abolished FA^11^, however, the mechanism how *Per2* may affect FA is unclear. Here we find that liver-specific deletion of *Per2* can inhibit FA by interfering with β-hydroxybutyrate (βOHB) production and its subsequent processing in the brain.

## Results

### Generation of conditional *Per2* ko mice

**Figure 1:**
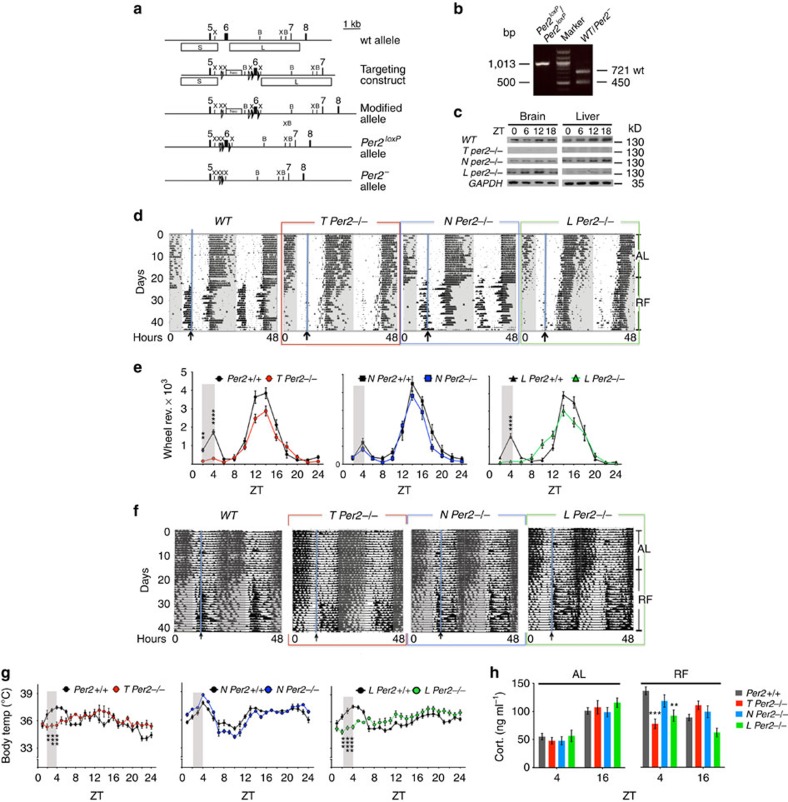
Mice with loss of *Per2* in the liver lack food anticipation. (**a**) Scheme illustrating the generation of a conditional *Per2* allele. For details see methods section. Numbers indicate exons, X, *Xho*I; B, *Bam*HI; neo, neomycin resistance; open triangles, *FRT* recombination sites; filled triangles, *loxP* recombination sites; box S, short arm of homology; box L, long arm of homology. (**b**) PCR products representing the *Per2*^*loxP*^, the wild-type (wt) and *Per2*^*−*^ alleles. (**c**) Western blot detecting PER2 protein in brain and liver tissue of the investigated genotypes. GAPDH as control. (**d**) Examples of double plotted wheel-running actograms under ad libitum (AL) and restricted feeding (RF) conditions. The arrow and blue line delineate daily access to food. (**e**) Quantification of wheel-running activity of *n*=10–16 animals for each genotype. The grey area highlights the time of anticipatory activity. (**f**) Examples of double plotted body temperature measurements under AL and RF conditions. The arrow and blue line delineate the start time of daily access to food. (**g**) Quantification of body temperature profiles of *n*=6 animals for each genotype (**h**) Corticosterone levels in the plasma of depicted genotypes under AL and RF conditions with *n*=4 for each genotype. All values are mean±s.e.m. For all analysis two-way ANOVA with Bonferroni *post hoc* test was applied. **P<0.01, ***P<0.001, ****P<0.0001. ANOVA, analysis of variance.

A major gap in knowledge of circadian biology is where the oscillators that generate FA rhythms are located and how they work mechanistically. To close at least part of this gap, we generated mice with a conditional *Per2* allele to delete this gene in a cell–type-specific manner. We flanked exon 6 of the *Per2* gene with *loxP* sites to delete exon 6 following administration of Cre recombinase (Fig. 1a,b). The deletion leads to a truncated PER2 protein consisting of the first 188 N-terminal amino acids (out of 1,257) plus 23 unrelated amino acids containing no structural elements involved in interaction with other clock proteins. Hence it is highly unlikely that this strongly shortened protein interferes significantly with the clock mechanism, however, we cannot exclude potential side effects. To determine whether a *Per2*-dependent oscillator that can generate FA is located centrally in neurons of the brain or peripherally in hepatocytes of the liver, we crossed *Per2*^*loxP*^ animals with mice carrying a *Cre recombinase* transgene under the control of the nestin promoter (*Nes-cre*) or the albumin promoter (*Alb1-cre*), respectively. For comparison, a *Cre recombinase* transgene under the control of the cytomegalovirus promoter (*CMV-cre*) was used to delete *Per2* in the entire organism. Western blot analysis revealed that these total knock-out (ko) animals designated as *T Per2*^*−/−*^ lack PER2 protein in brain as well as in liver tissue over the 24 h of a day (Fig. 1c, GAPDH control for every individual strain is shown in Supplementary Fig. 1a). In neuronal ko mice (*N Per2*^*−/−*^) and liver ko mice (*L Per2*^*−/−*^) most of the protein is absent in brain but not liver and liver but not brain, respectively (Fig. 1c). The residual expression of Per2 in *N Per2*^*−/−*^ animals stems from *Per2* expression in astrocytes and other cells not expressing nestin (see Supplementary Fig. 1b for immunohistochemical analysis). Residual Per2 expression does not overlap with the neuronal marker NeuN in *N Per2*^*−/−*^ animals in various brain regions and deletion of *Per2* in neuronal cells does not affect clock gene expression in the liver (Supplementary Fig. 1c).

### Total and liver-specific *Per2* ko animals lack FA

To test FA, we subjected mice of the three genotypes and the respective littermate control (wild type (WT)) animals to a restricted feeding (RF) schedule in which they received meals at zeitgeber time (ZT) 4 containing 70% of the calories consumed under ad libitum (AL) conditions. Under these conditions all genotypes displayed a similar amount of wheel-running activity for both constant darkness (DD) as well as LD (Supplementary Fig. 2a). Prolonged DD led eventually to arrhythmic wheel-running behaviour in *T Per2*^*−/−*^ mice (Supplementary Fig. 2b). In RF conditions, WT as well as *N Per2*^*−/−*^ animals showed a bout of wheel-running activity before feeding time at ZT4 (Fig. 1d,e) referred to as food-anticipatory activity (FAA). Surprisingly, FAA was strongly reduced or absent in *L Per2*^*−/−*^ animals (Fig. 1d,e) comparable to the *T Per2*^*−/−*^ mice that showed lack of FAA as observed in *Per2*^*Brdm1*^ mutants^11^. Another parameter for FA, raise in body temperature before the predicted meal, was observed in WT and *N Per2*^*−/−*^ mice, but not in *L Per2*^*−/−*^ and *T Per2*^*−/−*^ animals (Fig. 1f,g). Similar observations were made under constant darkness conditions (Supplementary Fig. 2c,d). Corticosterone, a third parameter for FA, was similar under AL conditions in all genotypes but was reduced under RF at ZT4 in the plasma of *L Per2*^*−/−*^ and *T Per2*^*−/−*^ animals compared with *N Per2*^*−/−*^ and WT (Fig. 1h). In contrast, plasma glucose was comparable in all genotypes under AL as well as under RF conditions (Supplementary Fig. 2e). To control for neuron-specific deletion of *Per2* in the *N Per2*^*−/−*^ mice, we performed immunohistochemistry in the SCN (Supplementary Fig. 1) and determined period length and light resetting properties, both characteristic parameters of the clock that are dependent on a functional SCN. We find that circadian period is shortened in *N Per2*^*−/−*^ and *T Per2*^*−/−*^ mice (Supplementary Fig. 2f) as described for *Per2*^*Brdm1*^ mutants^14^ but *L Per2*^*−/−*^ animals had a normal period comparable to WT (Supplementary Fig. 2f). Also resetting of the clock by a nocturnal light pulse at CT14 was reduced in *N Per2*^*−/−*^ and *T Per2*^*−/−*^ mice (Supplementary Fig. 2g), as described for *Per2*^*Brdm1*^ mutants^15^. Resetting in *L Per2*^*−/−*^ animals was not affected (Supplementary Fig. 2g). These results indicate that light- and food-entrainable circadian rhythms can be functionally separated and that *Per2* in the liver is specific for FA.

### Liver *Per2* is necessary but not sufficient for FA

**Figure 2:**
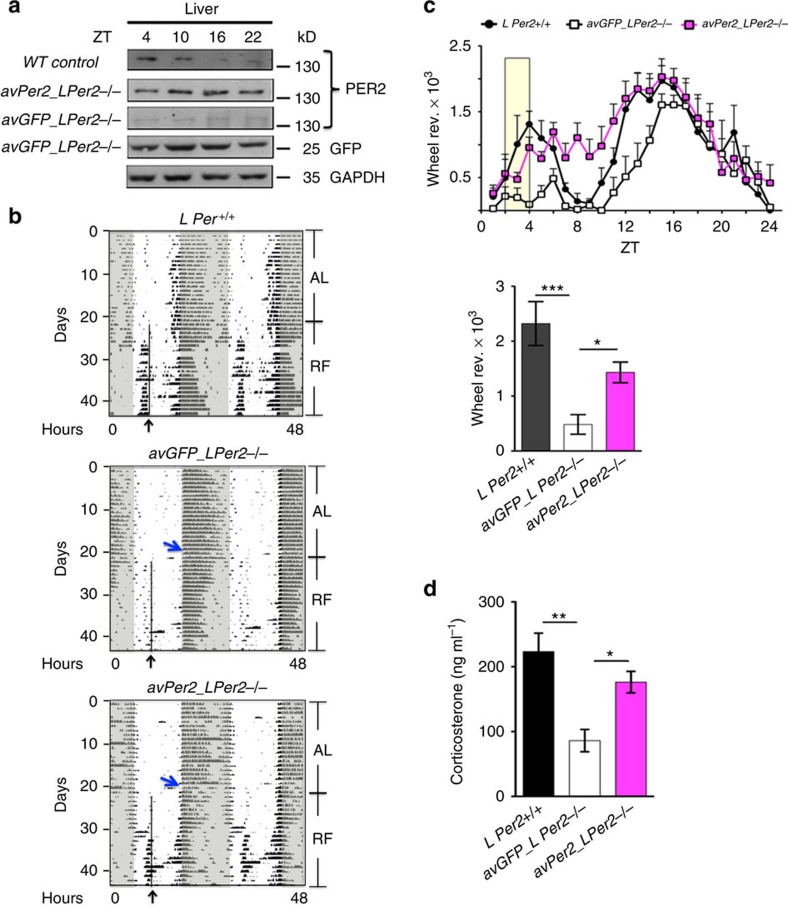
Rescue of food anticipation in *L Per2*^*−/−*^ mice. (**a**) Western blot of liver extracts from mice held under restricted feeding conditions showing constant expression of PER2 in the liver of *LPer2*^*−/−*^ mice after application of adenovirus expressing *Per2*. (**b**) Examples of double plotted wheel-running actograms under ad libitum (AL) and restricted feeding (RF) conditions. The black arrow and line delineate daily access to food. The blue arrow indicates adenovirus injection. *avGFP*, control adenovirus expressing green fluorescent protein (GFP). *avPer2*, adenovirus expressing *Per2*. (**c**) Quantification of wheel-running activity of *n*=6 animals for each genotype. Lower panel shows quantification of data from ZT2 to ZT4 (yellow area in upper panel). (**d**) Corticosterone levels in plasma (*n*=4). All values are mean±s.e.m. One-way ANOVA with Tukey's multiple comparison test, *P<0.05, **P<0.01, ***P<0.001. ANOVA, analysis of variance.

To verify whether liver *Per2* is necessary for FA, we injected an adenovirus expressing either *Per2* (*avPer2*) or as control green fluorescent protein (GFP) (*avGFP*) into *L Per2*^*−/−*^ and *T Per2*^*−/−*^ animals. To accomplish liver-specific overexpression of *Per2* or *GFP*, viruses were injected into the animal's tail vein where the virus passed through and then infected the liver specifically (Fig. 2a, Supplementary Fig. 3a). We observed that *avPer2* but not *avGFP* rescued FAA in *L Per2*^*−/−*^ animals (Fig. 2b,c), albeit with a delayed onset and reduced amplitude of FAA. Consistent with this observation, plasma corticosterone levels were reaching almost normal levels (Fig. 2d). In contrast *avPer2* did neither rescue FAA (Supplementary Fig. 3b,c) nor corticosterone levels (Supplementary Fig. 3d) in *T Per2*^*−/−*^ mice, indicating that liver *Per2* is necessary but not sufficient for FA. We conclude that *Per2* expression in at least one other tissue in addition to the liver is necessary for FAA and that the food-anticipatory oscillator is of systemic nature. The liver, however, seems to be important to generate a signal transmitting FA to the brain.

### Liver-specific deletion of *Per2* affects ketone body synthesis

**Figure 3:**
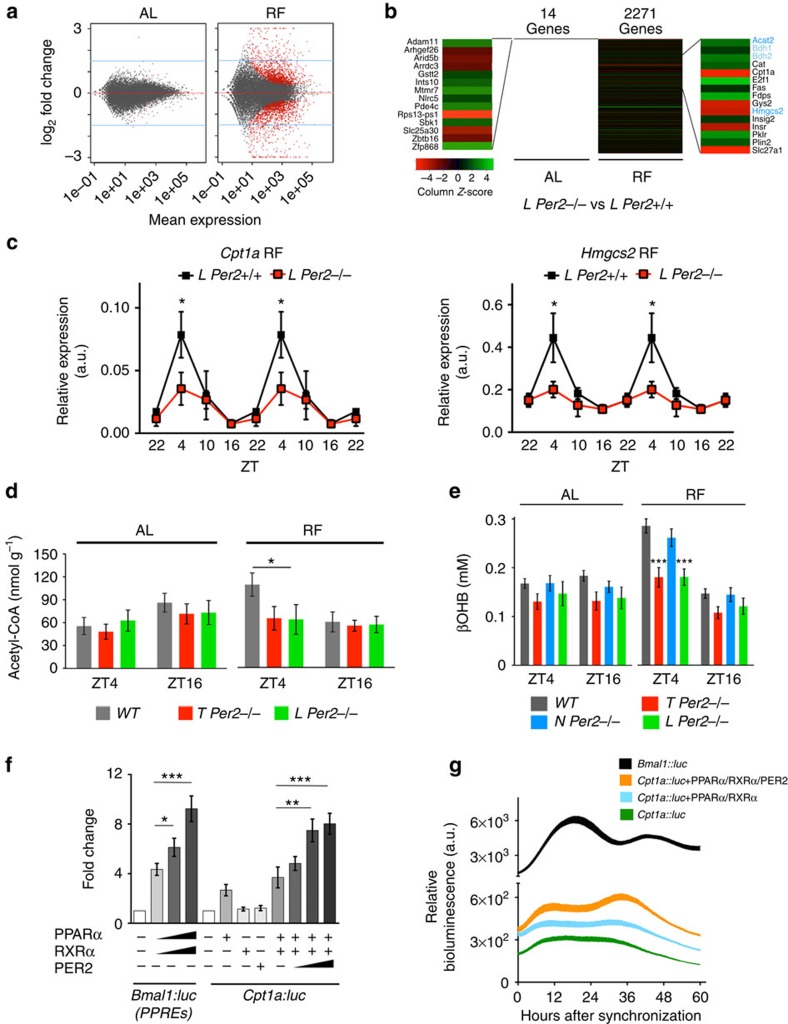
RNA-sequencing analysis of *L Per2*^*−/−*^ and control livers. (**a**) Comparison of *L Per2*^*−/−*^ (red) and control *L Per2*^*+/+*^(grey) mRNA under ad libitum (AL) and restricted feeding (RF) conditions. (**b**) Fourteen genes are different under AL and 2,271 genes under RF. Genes of fatty acid (black) and ketone body (blue) metabolism are indicated in the most right panel. (**c**) Quantitative PCR analysis of carnitin-palmitoyl transferase 1a (*Cpt1a*) and hydroxymethyl-glutaryl CoA synthase 2 (*Hmgcs2*) in liver tissue (*n*=6). Two-way ANOVA with Bonferroni post test. (**d**) Acetyl-CoA levels in liver extract (*n*=8). One-way ANOVA with Tukey's post test. (**e**) β-hydroxybutyrate (βOHB) levels (*n*=6). Two-way ANOVA with Bonferroni post test. (**f**) Transactivation assay in NIH 3T3 cells where *Cpt1a::luc* reporter was cotransfected with *Pparα*, *Rxrα* and increasing amount of *Per2* expression vectors (*n*=3). One-way ANOVA with Bonferroni post test. (**g**) Real-time monitoring of a *Cpt1a::luc* over several days alone or together with expression vectors for *Pparα*, *Rxrα* and *Per2.* The line thickness represents the data from three experiments with three replicas each. All values are mean±s.e.m. *P<0.05, **P<0.01, ***P<0.001. ANOVA, analysis of variance.

To understand how liver *Per2* may regulate FA, we performed RNA-sequencing experiments comparing mRNA expression at ZT4 of livers from *L Per2*^*−/−*^ and control littermates (*L Per2*^*+/+*^). At this time point, *Per2* expression is very low under AL conditions and therefore it is not astonishing that only 14 genes differed in expression between the 2 genotypes. In contrast, under RF conditions this difference rose to 2,271 genes (Fig. 3a,b). Of particular interest to us were genes involved in catabolic processes of fatty acids, as fatty acids serve as fuel following a period of fasting as experienced by mice under RF. We identified *carnitine palmitoyl transferase 1a* (*Cpt1a*) and *hydroxy-methlglutharyl-CoA synthase 2* (*Hmgcs2*), two mitochondrial enzymes that show reduced mRNA expression levels at ZT4 in *L Per2*^*−/−*^ mice under RF conditions (Fig. 3c) but not under AL conditions although the diurnal expression is still maintained with peaks at ZT16 and ZT10, respectively (Supplementary Fig. 4a,b). Cpt1a catalyses the rate-limiting step of the transfer of long-chain fatty acids from the outer to the inner mitochondrial membrane where they are liberated as acyl-CoA. β-oxidation results in acetyl-CoA that can either enter the Krebs cycle or be used by Hmgcs2 for the production of ketone bodies such as βOHB. In line with the reduced expression of *Cpt1a* and *Hmgcs2* in the liver of *L Per2*^*−/−*^ mice, we observed reduced acetyl-CoA as well as βOHB levels in the plasma of these and *T Per2*^*−/−*^ animals under RF but not under AL conditions (Fig. 3d,e). In contrast βOHB levels were not affected in *N Per2*^*−/−*^ mice (Fig. 3e). Interestingly, adenovirus-mediated expression of *Per2* in the livers of *L Per2*^*−/−*^ and *T Per2*^*−/−*^ animals rescued βOHB levels in the plasma (Supplementary Fig. 4c,d) and to a lesser extent also acetyl-CoA levels in liver extracts (Supplementary Fig. 4e,f), strongly suggesting an involvement of PER2 in the regulation of the rate-limiting enzymes of these two metabolites. Analysis of the transcriptional architecture and chromatin landscape of the circadian clock in mice revealed potential binding of the PER2 protein to the promoter of *Cpt1a*^16^. We find that PER2 regulates a *Cpt1a::luc* reporter in cell culture in a dose-dependent manner (Fig. 3f) in the presence of RXRα and PPARα, which binds to PER2 (ref. 17). This regulation by PER2 seems to be also time dependent, as revealed by real-time monitoring of the *Cpt1a::luc* reporter (Fig. 3g). The *Hmgcs2* promoter, however, appears to be indirectly affected by *Per2* through an unknown mechanism. Taken together it is reasonable to propose that *Per2* regulates a signal generated in the liver by directly modulating *Cpt1a* and indirectly affecting *Hmgcs2* expression. This signal may be βOHB, a ketone body that has been proposed as a signalling metabolite^18^.

### Timed release of βOHB partially rescues FA

**Figure 4:**
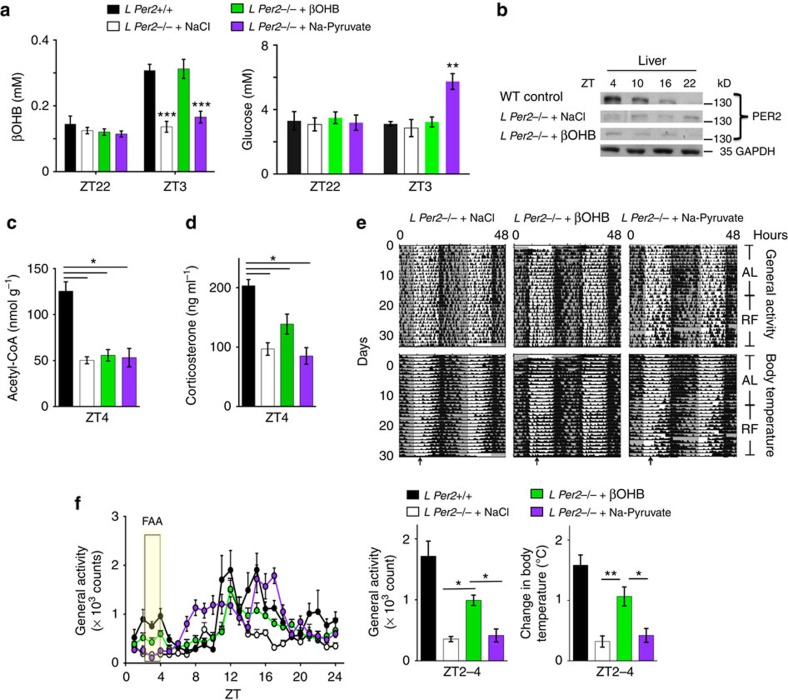
Rescue of food anticipation in *L Per2*^*−/−*^ mice by β-hydroxybutyrate. (**a**) Timed release of βOHB (green) but not NaCl (white) or Na-Pyruvate (purple) in *L Per2*^*−/−*^ mice mimics the βOHB levels in plasma of *L Per2*^*+/+*^ control animals (black). Measured after 15 days of infusion. (**b**) Western blot showing PER2 levels in the liver after application of NaCl and βOHB, respectively. (**c**) Acetyl-CoA levels in liver extracts of βOHB treated (green), NaCl-treated (white), Na-Pyruvate treated (purple), and control animals (black). (**d**) Corticosterone levels in plasma of βOHB treated (green), NaCl-treated (white), Na-Pyruvate treated (purple), and control animals (black). (**e**) Examples of double plotted activity and body temperature profiles of NaCl-treated, βOHB-treated, and Na-Pyruvate treated, *L Per2*^*−/−*^ mice. Arrows indicate food access. (**f**) Left: quantification of the activity profile (*n*=4–6), yellow rectangle represents ZT2–4, 2 h prior to food access. Right: quantification of activity and body temperature, respectively, before food access (*n*=4–6). All values are mean±s.e.m. One-way ANOVA with Tukey's post test. *P<0.05, **P<0.01, ***P<0.001. ANOVA, analysis of variance.

To test the potential of βOHB as a FA signal, we utilized programmable mini-pumps that released βOHB s.c. at ZT22 to reach a concentration normally observed in WT mice under RF preceeding feeding time (Supplementary Fig. 5b). As controls we used sodium pyruvate and coconut oil as a source of glucose and medium/short chain fatty acids, respectively. Both substances deliver precursors for acetyl-CoA that can enter mitochondria independently of Cpt1a. At ZT3, βOHB levels but neither glucose nor free fatty acid levels increase in control animals under RF conditions (black bars Fig. 4a and Supplementary Fig. 5c). Upon timed delivery of Na-pyruvate and coconut oil, glucose and free fatty acid levels increased, respectively (Fig. 4a and Supplementary Fig. 5c). Application of βOHB did neither induce PER2 levels nor increase acetyl-CoA levels in the liver (Fig. 4b,c, Supplementary Fig. 5d). Interestingly, however, plasma corticosterone levels were increased (Fig. 4d). Application of Na-pyruvate had no effect on both the acetyl-CoA and corticosterone levels (Fig. 4c,d). In accordance with these observations, timed application of βOHB but not saline nor Na-pyruvate nor coconut oil rescued FAA and anticipatory temperature increase in *L Per2*^*−/−*^ mice (Fig. 4e,f, Supplementary Fig. 5c) to a large extent, indicating that βOHB can serve as a signal for FA even in the absence of liver *Per2*. However, the partial rescue indicates that liver *Per2* is probably involved in the generation of an additional signal which may act together with βOHB to increase corticosterone levels, and rescue FA to its full extent.

## Discussion

FA needs a high degree of coordination between the metabolic needs of an organism and initiation of behaviour to seek for food that is available at a specific time of the day. Therefore, it was proposed that the circadian clock is part of this process. Since deletion of the SCN^7^ as well as deletion of certain clock components^9^ did not affect FAA it was postulated that a food-entrainable oscillator (FEO) not involving the SCN exists. This FEO appears not to rely on the circadian clock mechanism^9^.

*Per2* is not only part of the circadian oscillator, but can serve as a sensor for environmental signals such as light and food^3^,^15^,^19^. In addition, the PER2 protein interacts with nuclear receptors^17^ and thereby can influence a number of physiological processes. Hence, PER2 may act in the FEO as an inducer of effector signals in response to metabolic and sensory information rather than as a component of the circadian clock mechanism. Interestingly, however, we and others observed that total deletion of both *Per1* and *Per2* function does not affect FAA^9^,^20^,^21^, indicating that physiological functions regulated by *Per1* somehow interfere with functions regulated by *Per2*. This interference may be of systemic nature and it remains to be seen whether specific deletions of both genes in the liver affect FAA or not. Although a total deletion of *Per2* compared with the liver-specific deletion affects corticosterone levels in a similar way at ZT4 under RF conditions (Fig. 1h), the corticosterone levels of these two genotypes are not identical at ZT16, highlighting potential systemic effects of lack of *Per2* in the total ko. This is probably also related to the differences seen in wheel-running activity between the two genotypes at ZT8–12 (Fig. 1e).

Overexpression of *Per2* in the liver does not need to display a diurnal pattern to rescue FA in the *L Per2*^*−/−*^ mice (Fig. 2). Interestingly, this overexpression does also not affect diurnal *Bmal1* and *Per1* levels in the liver (Supplementary Fig. 6), suggesting that on RF PER2 may be uncoupled from the clock mechanism as proposed previously^22^. Importantly, liver rescue of *Per2* expression does not normalize FAA in the *T Per2*^*−/−*^ animals (Supplementary Fig. 2), indicating that the presence of *Per2* in other tissues is necessary. Since the increase in corticosterone levels in response to RF were not observed in *T Per2*^*−/−*^ animals (Supplementary Fig. 3d), the presence of *Per2* in the hypothalamic-pituitary-adrenal axis (HPA-axis) is probably important. This is consistent with previous findings showing that deletions in various brain regions affect FAA at least partially^8^. Hence, the FEO is probably of systemic nature.

Our study indicates that liver *Per2* is important for generating one or more signals to induce FAA. We propose that such a signal may be βOHB, a ketone body that has been suggested to serve as a signalling metabolite. βOHB signals via extracellular receptors and acts as an endogenous inhibitor of histone deacetylases thereby influencing gene expression via chromatin modifications^18^. Interestingly, constant overexpression of *Per2* in the liver can rescue FA in *L Per2*^*−/−*^ mice (Fig. 2) but only timed but not mistimed release of βOHB does the same (Fig. 4, Supplementary Fig. 7), indicating that the timing of βOHB release may be regulated by systemic cues. Because the rescue of FA after applying βOHB to *L Per2*^*−/−*^ mice is only partial (Fig. 4f), additional signals either from the liver and/or other organs most likely exist. One candidate is ghrelin, which is secreted by oxyntic cells in the stomach in anticipation of a regularly scheduled meal time. This activates ghrelin receptors on NPY/AgRP neurons promoting feeding behaviour^23^. In line with this mechanism is the observation that ablation of AgRP neurons impairs adaptation to RF^24^. Interestingly, ghrelin affects clock phase in the SCN and advances wheel-running behaviour following food deprivation^25^, indicating that ghrelin may regulate FAA. Mice-lacking ghrelin receptors display reduced FAA, suggesting that ghrelin contributes to FAA^23^. Furthermore, stomach ghrelin-secreting cells co-express ghrelin and the clock proteins PER1 and PER2 in a circadian manner^26^. Therefore these cells have been proposed to be part of the FEO. Oxyntomodulin, a hormone released by the gut after a meal, can reset the liver clock^27^ and thereby potentially affect ketone body production. It will be interesting to see in future experiments to what extent *Per2* is necessary in the gut and stomach to regulate oxyntomodulin and ghrelin, respectively, to test their role in FAA.

Time-RF can stabilize and reverse the progression of metabolic disease in mice with pre-existing obesity and type II diabetes^28^. Since βOHB levels rise in response to RF to induce activity for food uptake leading to prevention of acidosis, βOHB may be used as a timing signal to synchronize metabolism. This would be beneficial to accelerate adaptation to jet-lag. However, controlling the levels of βOHB will be critical, since too much of it may induce acidosis.

**Figure 5:**
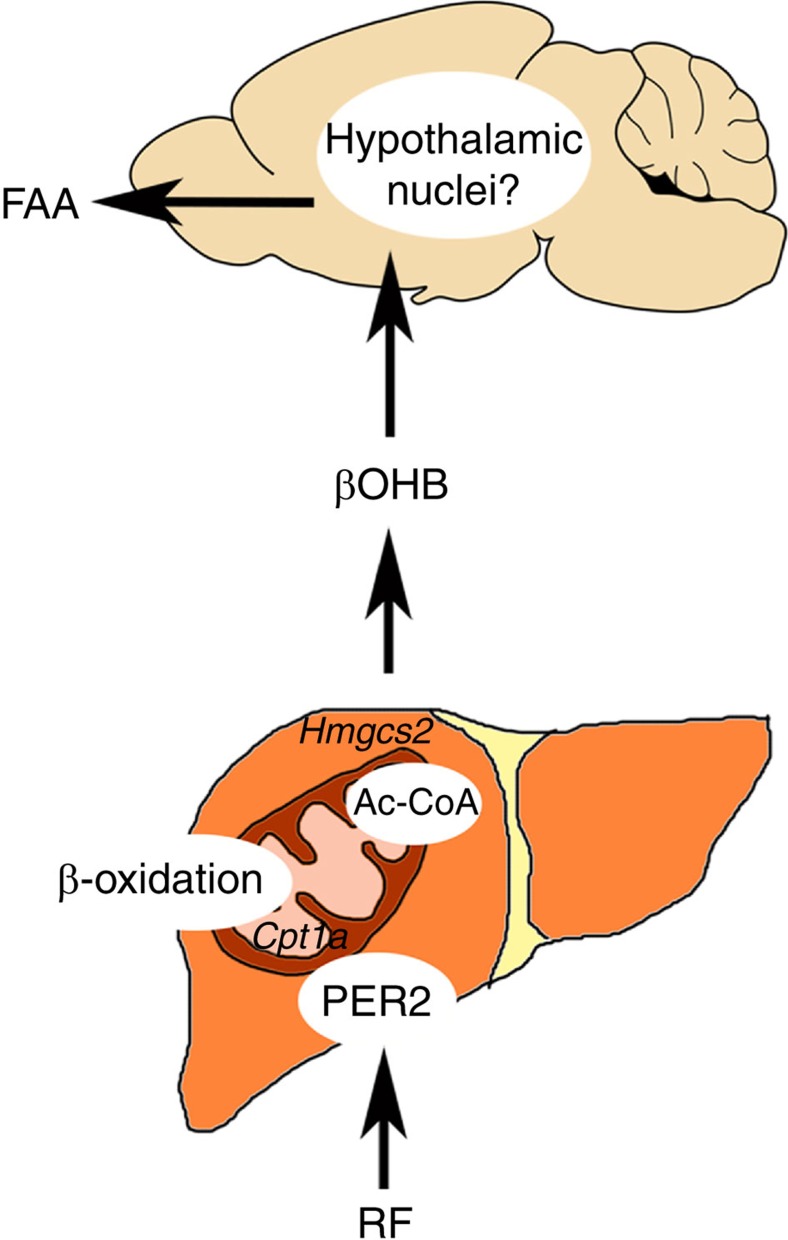
Regulation of FAA by Per2. Under RF conditions, liver *Per2* modulates *Cpt1a* and *Hmgcs2* expression, rate-limiting enzymes for βOHB synthesis. βOHB in the bloodstream reaches the brain and probably acts on hypothalamic nuclei and other brain cells. This activates the HPA-axis leading ultimately to food-anticipatory activity. The current study implies the necessity but not sufficiency of *Per2* in liver for FAA. Ac-CoA, acetyl-coenzyme A; Cpt1a, carnitine palmitoyltransferase 1A; FAA, food-anticipatory activity; Hmgcs2, 3-hydroxy-3-methylglutaryl-CoA synthase 2; RF, restricted feeding; βOHB, β-hydroxybutyrate.

Taken together our data indicate that under limited food availability, liver *Per2* regulates *Cpt1a* and indirectly also *Hmgcs2*, thereby increasing βOHB production, which then serves as a signal to make the animal anticipate feeding time (Fig. 5). Hence, information on feeding time is converted by the liver into a metabolic signal, probably affecting not a single, but multiple regions in the brain. The animal consequently anticipates and prepares for food uptake, which may be the advantage of initiating such behaviour. Interestingly, the establishment of FA takes a couple of days indicating that the receiver side, the brain, has to synchronize or metabolically adapt to the signal. For this process, *Per2* in the liver is necessary. However, *Per2* in the liver is insufficient by itself, suggesting that FA requires the expression of *Per2* in additional tissues, supporting the view that the food-anticipatory oscillator is of systemic nature.

## Methods

### Generation of conditional Per2 mice (Per2^loxP^)

A cassette containing *neomycin acetyltransferase (neo)* flanked by two FRT sites^29^ was inserted into a bacterial artificial chromosome encompassing the *Per2* locus (clone bMQ-293D21). Two loxP sites flanking exon 6 of *Per2* (which is coding for the PAS-A domain) were introduced to delete exon 6 and putting exon 7 out of frame leading to a truncated PER2 protein consisting of 188 amino acids plus 23 unrelated amino acids that is unstable and non-functional. The neo-cassette for selection in embryonic stem cells was between exon 5 in the short arm of homology (2.1 kb) and the loxP site upstream of exon 6. The loxP site downstream of exon 6 was followed by the long arm of homology (4.1 kb) containing exon 7.1 × 10^7^. ES cells (HM-1) were electroporated with the *SacI* and *SnaBI* linearized targeting vector using standard methods. Gene targeting was performed by PolyGene (Rümlang, Switzerland). Homologous recombination was verified using a primer just outside the short arm of homology of the targeting vector (5′-CCCAAGCAGAAAGCAGGTAGTC-3′) and a primer in the neo-cassette (5′-CAGCAGCCTCTGTTCCACATAC-3′) resulting in an amplicon of 2.8 kb. Recombination on the long arm of homology was verified by long-range PCR using a primer within the neo-cassette (5′-GTTGTGCCCAGTCATAGCCGAATAG-3′) and primer binding to the *Per2* locus that is not contained in the targeting vector (5′-CCCTTCCTGCCTTTCTCTTGA-3′) resulting in an amplicon of 5.5 kb. C57Bl/6 female mice (Charles River, WIGA Sulzfeld) were superovulated using standard procedures and mated with C57Bl/6 breeder males (Charles River). Blastocysts were injected with ES cell clones and transferred into pseudopregnant B6CBAF1 females (Polygene, originally obtained as inbred strains from Harlan Laboratories). Male chimeras were bread with *Flp* deleter mice to remove the *neo*-cassette^30^. Agouti offspring were tested for germline transmission of the targeted *Per2*^*loxP*^ allele using the following primers: E6UP 5′-CTGTGTCCCTGGTTTCTG-3′ and Cre2 5′-GCAGGGCAGTTTCATCAAGG-3′ resulting in amplicons of 468 bp for WT and 596 bp for the transgene. The deletion of the FRT flanked *neo* cassette was tested by PCR using the following primers: E6DOWN 5′-AGGGCGGAAGCTTGTAAGGG-3′ and Cre1 5′-AGCTGGCAAAGGTCACTC-3′ resulting in amplicons of 417 bp for WT and 623 bp for the transgene.

### Generation of tissue-specific Per2 ko animals

Mice with a loss of *Per2* in all body cells were generated by breeding mice with the conditional *Per2* allele (*Per2*^*loxP*^) with mice carrying a *Cre recombinase* transgene under the control of the cytomegalovirus promoter (*CMV-cre*) (European Mouse Mutant Archive (EMMA) EM:01149, B6.129-Cre-Deleter: B6.C(129)-Tg(CMV-cre)1Cgn/CgnIbcm, received from the Mouse Genetics Cologne Stiftung (MGC Foundation))^31^.

Mice with loss of *Per2* in most cells of the central nervous system were generated by breeding mice with the conditional *Per2* allele (*Per2*^*loxP*^) with mice carrying a *Cre recombinase* transgene under the control of the nestin promoter (*Nes-cre*) (EMMA) EM:04561, Bclaf1 × Tg Nes-cre C57BL/6: Tg(Nes-cre)1Kln, received from the FMP Leibnitz-Institute für Molekulare Pharmakologie)^32^.

Mice with loss of *Per2* in hepatocytes were generated by breeding mice with the conditional *Per2* allele (*Per2*^*loxP*^) with mice carrying a *Cre recombinase* transgene under the control of the albumine promoter (*Alb1-cre*) (EMMA) EM:00603, STOCK Tg(Alb1-cre)7Gsc/Ibcm: Tg alfpCre, received from Consiglio Nazionale delle Ricerche EMMA Monterotondo)^33^.

Genotyping after Cre-mediated recombination was performed by PCR with the primers L1, 5′-AGCTGGCAAAGGTCACTC-3′ and L2, 5′-GCAGGGCAGTTTCATCAAGG-3′ resulting in products of 1,013 bp for *Per2*^*loxP*^, 721 for WT and 450 bp for *Per2*^*−*^.

All experiments described were performed with paired littermate mice (C57BL/6 x 129) carrying a single copy of the *Cre* transgene and homozygous either for the *Per2* conditional allele or for the WT *Per2* allele (controls). This arrangement controls for any potential phenotype caused by the persistent expression of the Cre recombinase protein. In the study male and female mice were used.

### Animal housing

All mice were housed in individual cages with or without running wheel in light and soundproof ventilated chambers. All mice were entrained to a 12:12-h LD cycle, where times of day were converted to ZTs (ZT0 lights-on). Housing as well as experimental procedures were performed in accordance with the guidelines of the Schweizer Tierschutzgesetz (TSchG, SR455) and the declaration of Helsinki. The protocol was approved by the state veterinarian of the Canton of Fribourg.

### Restricted feeding

In a first set of experiments, male and female littermate controls (*WT*), *T Per2*^*−/−*^*, N Per2*^*−/−*^*, L Per2*^*−/−*^mice (3–5 months old) were kept in individual cages with a wheel for 3 weeks having access to water and food AL. During acclimatization, daily food intake was determined. The mice were progressively challenged with RF for 3 weeks. Food restriction accomplished by limiting food access to 8 h a day (ZT4-ZT12), and by providing food 80% of their normal daily food intake in first week and 70% of their normal daily food intake in following 2 weeks. The body weight of mice was measured twice a week. Mice were re-fed consequently if any had lost more than 20% of its initial body weight.

In a second set of experiments, all *WT, T Per2*^*−/−*^*, N Per2*^*−/−*^ and *L Per2*^*−/−*^mice (3–5 months old) were placed in individual cages with a wheel, under AL condition and entrained to 12:12-h LD cycle. After 3 weeks, mice were subjected to DD condition, and were exposed simultaneously to RF condition (same as above) for the next 3 weeks.

### General activity and body temperature

Male and female *T Per2*^*−/−*^*, N Per2*^*−/−*^*, L Per2*^*−/−*^ mice and their corresponding littermate WT controls (3–5 months old) were kept in individual cages with no wheel and subjected to 12:12 h LD cycle. Under gaseous isoflourane anaesthesia, each mouse received a biotelemetry transmitter (G2 E-mitter, series 4,000 system; Starr Life Sciences Corp., USA) for monitoring core body temperature and general locomotor activity. The mice were subjected to RF condition same as described above.

### Light-induced phase resetting

Resetting experiments were performed according to *Aschoff type I* protocol. Male and female *T Per2*^*−/−*^*, N Per2*^*−/−*^and *L Per2*^*−/−*^ mice and their littermate controls (*WT*) (4–6 months old) were placed into separate wheel-running cages and entrained to a 12:12 LD cycle for 2 weeks, and released in constant DD, and after 10 days light pulse was given at CT14 (early subjective night). Wheel-running activity was recorded over the next 7 days. Following this period, mice were re-entrained to 12:12 h LD cycle for 3 weeks, and the procedure repeated except that mice received a phase-advancing light pulse at CT22 (late subjective night). The period length in DD was assessed on 10 consecutive days of stable free-running activity by *χ*^*2*^-periodogram analysis. Phase shift was determined by calculating distance between the first regression line fitting through at least six onsets of activity before, and the second regression line fitting through at least six onsets of activity after light pulse (first 2 days after light pulse were not considered for calculation of phase shift)^34^.

### Data acquisition and analysis

Wheel-running activity actograms were acquired, and analysed with Clocklab software (Actimetrics, USA). Mean daily activity was quantified for each genotype during last 7 days of RF protocol. Average wheel revolutions were quantified every 2 h, and mean activity profiles for each genotype during AL and RF periods can be assessed. The general activity and core body temperature were recorded for every minute with VitalView data acquisition system. General activity count and body temperature were averaged for every hour, so that mean daily general activity and body temperature profile can be examined in both AL and RF conditions. Wheel-running/general activity and change in body temperature 2 h prior to meal time (ZT2–ZT4) was quantified, which was designated as FAA^11^.The infusion profile was programmed to SMP/UCD 300 pump and monitored via iPRECIO IMS-300 V334 software.

### Tissue and blood sampling

All genotypes (*T Per2*^*−/−*^*, N Per2*^*−/−*^ and *L Per2*^*−/−*^, and their littermate *WT* controls) were housed individually and were killed during AL and RF conditions at different time points (ZT4, ZT10, ZT16 and ZT20). The liver and brain were isolated, and immediately frozen in liquid nitrogen and stored at −80 °C.

The blood was collected in EDTA-coated tubes, and centrifuged at 4,000 r.p.m. for 10 min to isolate plasma. The clear plasma was stored at −80 °C. For the virus-injected mice, thymus and kidney were collected along with the brain and liver. During all the sampling, mice were subjected to gaseous isoflurane anaesthesia.

### RNA extraction and library construction

Liver samples were immediately flash frozen in liquid N_2_ and stored at −80 °C. RNA was extracted using NucleoSpin RNA (Machery-Nagel, Düren, Germany) according to the instructions of the manufacturer. Quality of the RNA samples was analysed with a spectrophotometer, agarose gel electrophoresis and reverse transcription–PCR. Library construction starting from the poly(A)-tail and multiplexing was performed according to the instructions of the manufacturer (Illumina). The samples were organized as follows:

Three replicas (WN1, WN2, WN3) correspond to genotype WT (*L Per2*^*+/+*^) (normal feeding),

Three replicas (WR1, WR2, WR3) correspond to genotype WT (RF),

Three replicas (PN1, PN2, PN3) correspond to genotype *L Per2*^*−/−*^ (normal feeding), and

Three replicas (PR1, PR2, PR3) correspond to genotype *L Per2*^*−/−*^(RF).

For the experiment, complementary DNA (cDNA) libraries were barcoded using Illumina primers and loaded onto one lane of an IlluminaHS2000 machine. cDNA libraries were diluted and the 12–15 pmol of material were loaded onto each lane. Template hybridization, extension of the template, amplification of the template, cluster generation, sequencing primer addition and single-end sequencing were performed according to the instructions of the manufacturer. The samples were sequenced for a maximum sequencing length of 75 bp and their barcodes identified afterwards.

### Analysis of RNA-Seq data sets

Sequences were aligned to the mouse genome (UCSC version mm10 database). Numbers of the sequences obtained for each library can be found in Supplementary Table 1.

Sequences (fastq format) were mapped with Bowtie2 (ref. 35) and with Tophat^36^, though no ribosomal RNA removal has been performed. Uniquely mapped sequences from the output files (bam format) were then used for further analysis. Bam files (with corresponding index files) were used for signal visualization with the Integrative Genomics Viewer^37^, as demonstrated in Supplementary Fig. 8 for the control of the experiment. After visualization of all files, we counted the reads with HTseq-count^38^ using the following criteria:

samtools view sample_ID.bam | htseq-count-a 10-s no-i gene_name-Mus_Musculus.gtf>sample_ID_counts.txt

Tests for differential expression between the samples were performed in R software (R Core Team, 2014 http://www.R-project.org/) using the DESeq2 package^39^ (Supplementary Software). A threshold on the corrected *P* value was used to call for differentially expressed genes (*P*.adjust<0.05).

The analysis performed with Pathway studio (http://www.elsevier.com/online-tools/pathway-studio) was a complementary analysis to identify molecular interactions and involved pathways after obtaining data on the differential expression analysis between the two groups of the genotype. This software generates interaction information by analysing sentences extracted from the literature.

### RNA extraction and quantitative PCR analysis

Total RNA was extracted from frozen liver, and brain samples using RNA-Bee (AMS Biotechnology). RNA samples were treated with DNase I (Roche) and purified by phenol:chloroform extraction and ethanol precipitation. cDNA synthesis was carried out with SuperScript II (Invitrogen) and random priming. TaqMan probe-based real-time PCR was performed for mRNA quantification (KAPA PROBE FAST, KAPA Biosystems, RotorGene 6,000, Corbett Research). All RNA samples were normalized to *Gapdh.* All the Primers are listed in Supplementary Table 2

### Western blot analysis

Total proteins were extracted from frozen brain and liver in RIPA buffer (25 mM Tris–HCl pH7.5, 1 mM EDTA pH8.0, 1% Triton X-100, 0.5% Na-deoxycholate, protease inhibitor cocktail from Roche diagnostics). SDS–PAGE and immunoblot analysis were performed according to standard protocols. Proteins were separated by SDS–PAGE (6.5%) and transferred to nitrocellulose membranes. Membranes were incubated with anti-PER2 antibody (Alpha diagnostics, TX, USA) 1:2,000, anti-GFP (50430-2-AP, Proteintech, USA) and anti-GAPDH antibody (ab9483, abcam, UK) 1:2,000. Quantification was performed using the Quantity One analysis software (Bio-Rad). GAPDH was used for normalization. Original blots are shown in Supplementary Fig. 9.

### Adenovirus production

Recombinant adenoviruses were generated using the AdEasy technology^40^. A full-length mouse *Per2* cDNA (3,774 bp) was amplified by PCR using sense primer (5′-GAGATCTAGCCCCATGAATGGATACGTGGAC-3′; *BglII* site) and anti-sense primer (5′-CGTCGAGTTACGTCTGGGCCTCTATCCTG-3′; *XhoI* site), and cloned into pAdTrack-CMV shuttle vector. *Escherichia coli* BJ5183 cells containing the AdEasy backbone vector were transformed with linearized pAdTrack-*mPer2* to construct recombinant adenovirus. HEK293T cells were transfected with the recombinant adenoviral vectors, and recombinant adenovirus was amplified by several rounds of infection, and purified by AdEasy Virus Purification Kit (#240243, Agilent Technologies). The generated viruses were designated as *avGFP* and *avPer2*. The viruses were titrated with AdEasy Viral Titer Kit ((#972500, Agilent Technologies). Approximately 10^10^ viral particles were injected into the tail vein.

### Adenovirus injection

Male and female *T Per2*^*−/−*^*, L Per2*^*−/−*^ mice (3–5 months old) were placed in individual cages with wheel, having AL access to water and food. After 3 weeks of habituation, viral particles (pAdEasy-GFP (avGFP) or pAdEasy-Per2 (avPer2)) were injected via the tail vein to enable virus particles to reach the liver via the portal vein. The tail vein injection procedure was carried out under gaseous isoflurane anaesthesia, and the virus-infected mice were subjected to the RF protocol (same as described above).

### Plasma metabolic parameters

Blood samples were collected via tail vein, and plasma was recovered by centrifuging samples at 4,000 r.p.m. for 10 min. Plasma glucose, beta-hydroxybutyrate, free fatty acids, and corticosterone levels were measured with the Glucose (HK) Assay kit (Sigma-Aldrich, St Louis, USA), beta-hydroxybutyrate (beta HB) Assay kit (ab83390, abcam), Free Fatty Acid Quantification kit (ab65341, abcam) and Corticosterone EIA kit (ADI-900-097, Enzo Life Sciences, USA) respectively, according to the manufacturer's instructions.

### Hepatic acetyl-CoA measurement

Liver samples (100 mg) were deproteinized by pulverizing in 1 N perchloric acid (to avoid enzymatic interference in assay), and the supernatant was neutralized with 3 M KHCO3 (pH adjusted 6–8). This homogenate was used for acetyl-CoA measurement by using commercial assay kits according to manufacturer's protocols (ab87546, abcam).

### Cell culture, bioluminescence monitoring and luciferase assay

NIH 3T3 mouse fibroblast cells were used for *in vitro* experiments. Cells were maintained in Dulbecco's Modified Eagle Medium (DMEM), high glucose (4.5 g l^−1^) (6429, Sigma, USA) containing 10% foetal calf serum (FCS) and 100 U ml^−1^ penicillin/streptomycin at 37 °C in a humidified atmosphere containing 5% CO2.

Expression plasmids pSct1-PPARα, Psct1-RXRα, Psct1-PER2, Psct1-LacZ (β-galactosidase), pCMV SEAP (Secreted Alkaline Phosphatase) and Bmal1luciferase construct (with PPAR regulatory site) are described^17^.

For the bioluminescence assay, NIH 3T3 cells were transfected with Cpt1a luciferase reporter vector, pCMV SEAP for normalization and indicated expression vectors using linear polyethylenimine (Polysciences Europe) according to the manufacturer's instructions. Cells were synchronized by100 nM dexamethasone in DMEM. After 20 min, medium was replaced by phenol red-free DMEM supplemented with 5% foetal calf serum and 0.1 mM luciferine. Bioluminescence was recorded in real-time using a LumiCycle apparatus (Actimetrics). Transfection efficiency differences were corrected by normalization to the secreted alkaline phosphatase activity (SEAPactivity; Roche Applied Science). Bioluminescence data were analysed with LumiCycle analysis software (Actimetrics)^17^.

A 1,957 bp fragment of the mouse Cpt1a promoter region (−4,000 to −2,123 bp from the transcription start site containing *PPAR response elements*) was cloned into pGL3 basic vector (Promega, Madison, USA) using following primers: 5′-CGGTACCCCCTCTTCTAACCTG AG-3′ (sense primer; *KpnI* site) and 5′-GCTCGAGGTTACAGCGTGAGCCTGTC-3′ (anti-sense primer; *XhoI* site). Luciferase assays were performed in NIH3T3 cells as described^41^. An empty pGL3 vector and Bmal1:luc (with *PPAR response elements*) reporter were used as negative and positive controls, respectively.

### Mini-pump implant and infusion of βOHB

Male and female *L Per2*^*+/+*^ and *L Per2*^*−/−*^ mice (3–5 months old) were kept in individual cages with no wheel and subjected to 12:12 h LD cycle. Telemetry transmitter (G2 E-mitter) was i.p. implanted in each mouse under gaseous anaesthesia. At least 10 days after the transmitter implantation an iPRECIO programmable microinfusion pump (SMP/UCD 300; Primetech Corp., Japan) was implanted in subgluteal space (s.c. administration) on the back of each *L Per2*^*−/−*^mouse. Same anaesthesia procedure was followed as the one applied for transmitter implantation. After 1 week of surgery, the mice were exposed to scheduled feeding condition (as mentioned previously). The mice were infused with vehicle (saline) or βOHB (D-βOHB; 1.6 mmol kg^−1^ in saline, pH 7.4; Sigma-Aldrich)^42^ or sodium pyruvate (Na-pyruvate, 2 g kg^−1^, Sigma-Aldrich), or coconut oil (5 μl h^−1^, Sigma-Aldrich). The pumps were programmed to infuse the saline (2 μl h^−1^) or D-βOHB (2 μl h^−1^) or Sodium pyruvate (5 μl h^−1^) or coconut oil (5 μl h^−1^) prior to meal time (6 h, ZT22-ZT4) in RF. At the end of the infusion and RF period, the blood samples were collected in EDTA-coated tubes at different time points (ZT22, ZT3, ZT6 and ZT10) via tail vein to analyse βOHB, glucose and fatty acid levels in plasma.

### Immunohistochemistry

Animals used for the immunohistochemistry were killed at ZT12. Brains were perfused with 0.9% NaCl and 4% PFA. Perfused brains were cryoprotected by 30% sucrose solution and sectioned (20 μm, coronal) using cryostat. Sections chosen for staining were placed in 24-well plates (2 sections per well), washed three times with 1 × TBS and 2 × SSC (pH 7, 0.3 M NaCl/0.03 M tri-Na-citrate). Antigen retrieval was performed with 50% formamide/2 × SSC by heating to 65 °C for 50 min. Then, sections were washed twice in 2 × SSC and three times in 1 × T BS pH 7.5 (0.1 M Tris/0.15 M NaCl), before blocking them for 1.5 h in 10% normal donkey serum sterile (NDS, ab138579, Abcam, UK)/0.1% Triton X-100/1 × TBS at room temperature. After the blocking, primary antibodies anti-PER2 antibody 1:100, and Anti-NeuN antibody [1B7]-Neuronal Marker (ab104224) 1:400, diluted in 1% NDS/0.1% Triton X-100/1 × TBS were added to the sections and incubated overnight at 4 °C. Next day, sections were washed with in 1 × TBS and incubated with the appropriate fluorescent secondary antibodies diluted 1:500 in 1% NDS/0.1% Triton X-100/1 × TBS for 3 h at room temperature. (Alexa Fluor 488-AffiniPure Donkey Anti-Rabbit IgG (H+L) 711–545–152 and Alexa Fluor647-AffiniPure Donkey Anti-Mouse IgG (H+L) 715–605–150, all from Jackson Immuno Research). Finally the tissue sections were washed again twice in 1 × TBS and mounted on glass microscope slides. Fluorescent images were taken by using a confocal microscope (Leica TCS SP5), and images were taken with a resolution of 1,024 × 1,024, scan speed 200 hz with frame average 3. Images were processed with the LAS AS software from LEICA according to the study by Schnell *et al.*^43^

## Additional information

**Accession codes:** The RNA-seq data have been deposited in NCBI Gene Expression Omnibus database under accession code SRP058086.

**How to cite this article:** Chavan, R. *et al.* Liver-derived ketone bodies are necessary for food anticipation. *Nat. Commun.* 7:10580 doi: 10.1038/ncomms10580 (2016).

## Supplementary Material

Supplementary Information Supplementary Figures 1-9 and Supplementary Tables 1 & 2.

Supplementary Software R script used to analyze gene expression in RNA sequencing data.
