# Supplementary material for: Liver-derived ketone bodies are necessary for food anticipation
Source: Nat Commun. 2016 Feb 3;7:10580. doi: 10.1038/ncomms10580 (PMC4742855; doi:10.1038/ncomms10580)
Supplement: Supplementary Information — Supplementary Figures 1-9 and Supplementary Tables 1 & 2. [file ncomms10580-s1.pdf]

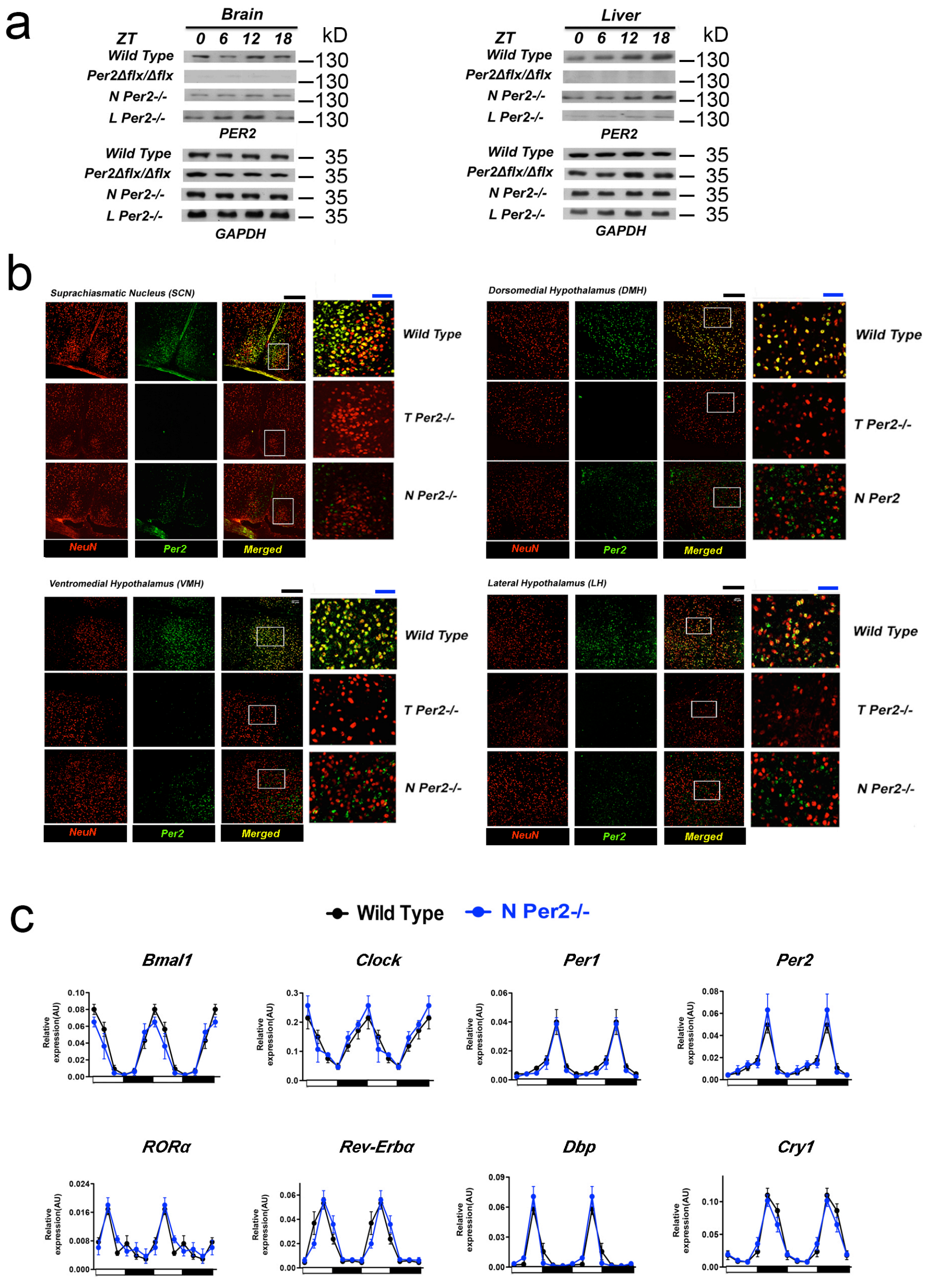

Supplementary Figure 1

Supplementary Figure 1 Validation of *Per2* deletion in neuronal cells in *N Per2*$^{-/-}$ mice. (**a**) Western blot from liver extracts of mice held under ad libitum conditions detecting PER2 protein in brain and liver tissue of the investigated genotypes. GAPDH as control for each genotype is shown in the panels to the right. (**b**)

Immunohistochemistry in the Suprachiasmatic nucleus (SCN), Dorsomedial hypothalamus (DMH), Ventromedial hypothalamus (VMH) and Lateral hypothalamus (LH) of wild type, *T Per2*$^{-/-}$ and *N Per2*$^{-/-}$ mice at ZT12. Antibodies recognizing NeuN (marker for differentiated neurons) and Per2 are in red and green, respectively. A white rectangle indicates enlarged part of merged image. Scale bar: 200 µm (black), 50 µm (blue). **(c)** Double-plotted (Mean ± SEM, n =3-4) mRNA levels of circadian oscillator components *Per2*, *Bmal1,Clock, Per1, Per2, ROR*α, *Rev-erb*α, *Cry1* and *Dbp* in liver of *N Per2*$^{-/-}$ mice under ad libitum condition at different times of the day. Transcript levels were measured by quantitative PCR and normalized to *Gapdh*.

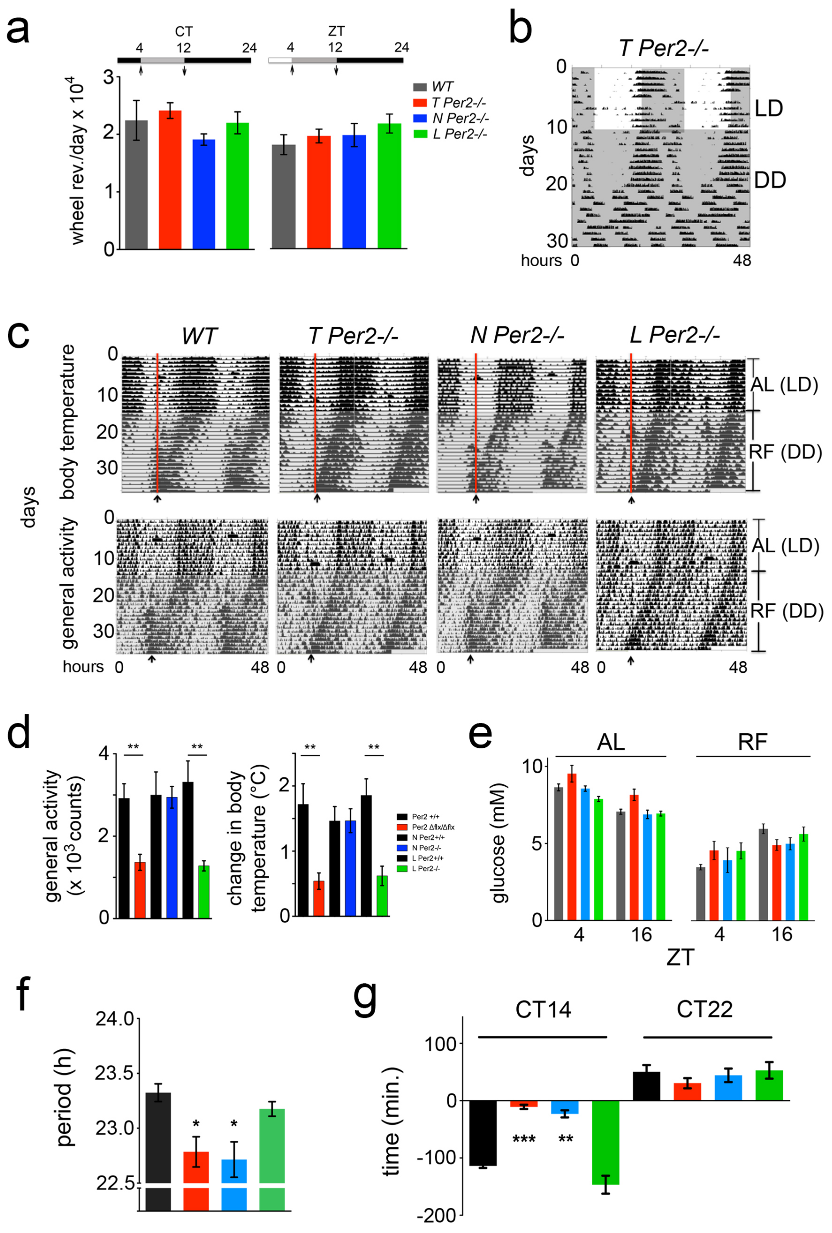

Supplementary Figure 2

Supplementary Figure 2 Characterization of mice. (**a**) Total daily wheel-running activity under constant darkness (DD) and 12 h/12 hours dark (LD) conditions of wild-type, *T Per2*$^{-/-}$, *N Per2*$^{-/-}$, and *L Per2*$^{-/-}$ mice (n = 5). Top bar indicates feeding schedule. (**b**) *T Per2*$^{-/-}$ animals become arrhythmic under prolonged DD conditions. (**c**) General activity and body temperature in DD conditions under AL and RF of all genotypes. (**d**) Quantification of general activity and change in body temperature in DD under RF conditions (n = 5). (**e**) Plasma glucose at ZT4 and ZT16 under AL and

RF conditions (n = 4-6). (**f**) Circadian period of all genotypes determined by χ^2-periodogram analysis (n = 6). (**g**) Quantification of phase shifts observed after a light pulse at CT14 and CT22 (n = 6). All values are mean ± SEM. One-way ANOVA with Tukey's multiple comparison test, $*p < 0.05$, $**p < 0.01$, $***p < 0.001$.

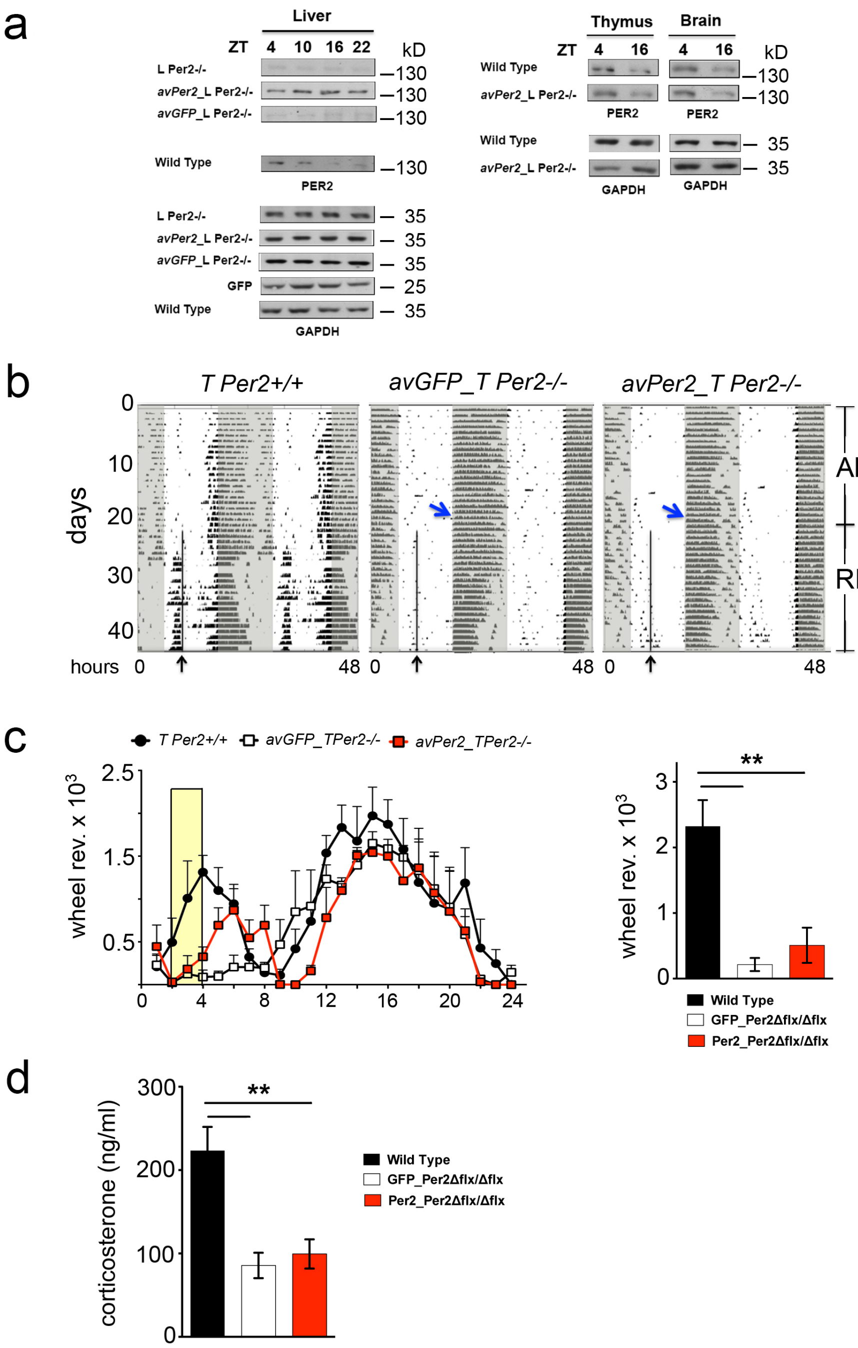

Supplementary Figure 3

Supplementary Figure 3 Lack of rescue of food anticipation in *T Per2*$^{-/-}$ mice by adenoviral overexpression of *Per2* in the liver. (**a**) PER2 protein in *avPer2* infected *L Per2*$^{-/-}$ mice in various tissues. GAPDH as control for each genotype and treatment (right). (**b**) Examples of double plotted wheel-running actograms under ad libitum (AL) and restricted feeding (RF) conditions. The black arrow and blue line delineate daily access to food. The blue arrow indicates start of RF. *avGFP* = control adenovirus expressing green fluorescent protein (GFP). *avPer2* = adenovirus

expressing *Per2*. (**c**) Quantification of wheel-running activity of n = 6 animals for each genotype. Right panel shows quantification of data from ZT2-ZT4 (yellow area, left panel). (**d**) Corticosterone levels in plasma (n = 4). All values are mean ± SEM. One-way ANOVA with Tukey's multiple comparison test, *p < 0.05, **p < 0.01, ***p < 0.001.

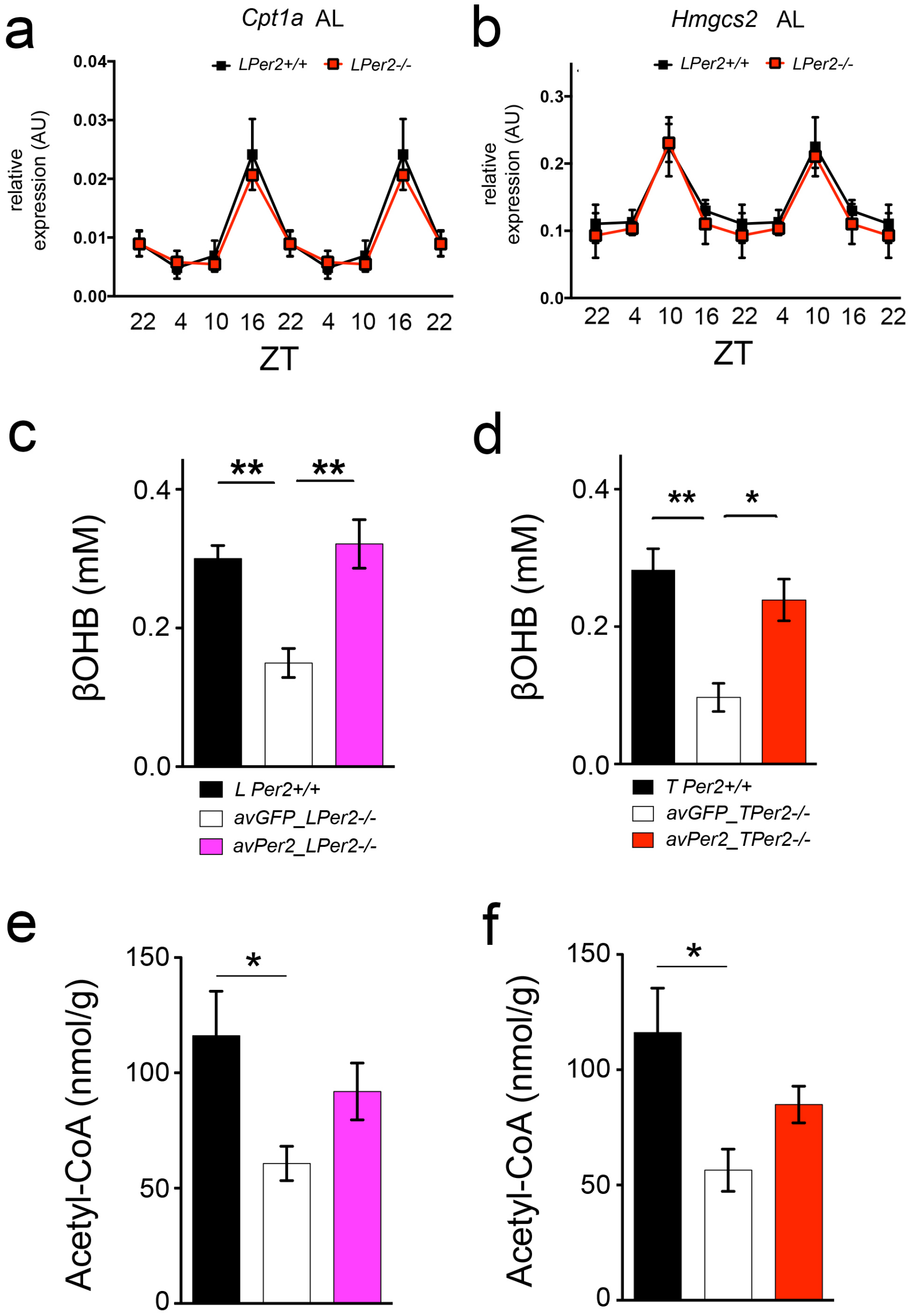

Supplementary Figure 4

Supplementary Figure 4 βOHB and acetyl-CoA levels after adenoviral Per2 and GFP expression in *L Per2*$^{-/-}$ and *T Per2*$^{-/-}$ mice. (**a**) and (**b**) Double plotted temporal expression profile of carnitin-palmitoyl transferase a (*Cpt1a*) and hydroxymethyl-glutaryl CoA synthase 2 (*Hmgcs2*) in liver tissue under ad libitum condition, respectively. Two-way ANOVA with Bonferroni multiple comparison test (n = 4-6) *p < 0.05, **p < 0.01 (**c**) βOHB plasma levels in *L Per2*$^{+/+}$ (black), avGFP_*L Per2*$^{-/-}$ (white) and avPer2_*L Per2*$^{-/-}$ mice (pink). (**d**) βOHB plasma levels in *T Per2*$^{+/+}$

(black), avGFP_*T Per2*$^{-/-}$ (white) and avPer2_*T Per2*$^{-/-}$ mice (red). (**e**) Acetyl-CoA levels in liver of *L Per2*$^{+/+}$ (black), avGFP_*L Per2*$^{-/-}$ (white) and avPer2_*L Per2*$^{-/-}$ mice (pink). (**f**) Acetyl-CoA levels in liver of *T Per2*$^{+/+}$ (black), avGFP_*T Per2*$^{-/-}$ (white) and avPer2_*T Per2*$^{-/-}$ mice (red). All values are mean ± SEM. One-way ANOVA with Tukey's multiple comparison test (n = 4), *$p < 0.05$, **$p < 0.01$.

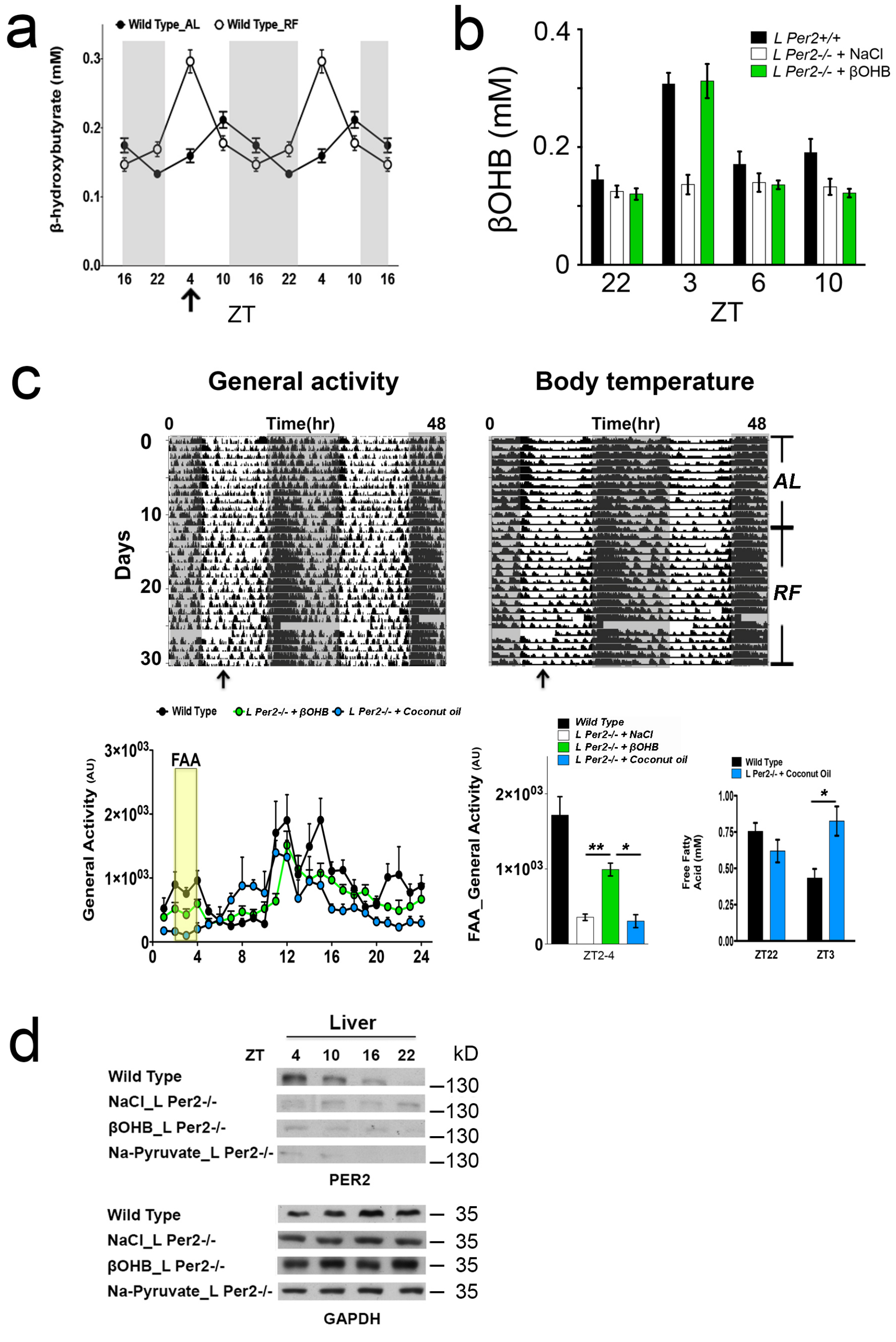

Supplementary Figure 5

Supplementary Figure 5 Temporal profile of βOHB in plasma and timed coconut oil application to mice. (**a**) Double-plotted plasma β-hydroxybutyrate (βOHB) levels (n = 5-8) in wild type mice under ad libitum (AL) and restricted feeding (RF) condition at different times of the day. Arrows indicate food access. (**b**) Timed release of βOHB (green) but not NaCl (white) in *L Per2*$^{-/-}$ mice mimics the βOHB levels in plasma of *L Per2*$^{+/+}$ control animals (black). Measured after 15 days

of infusion. (**c**) Top: Example of double plotted activity and body temperature actograms of coconut oil infused *L Per2*$^{-/-}$ mice under ad libitum (AL) and restricted feeding (RF) conditions. Arrows indicate food access. Bottom: quantification of the activity profile (n = 4-6), yellow rectangle represents ZT2-4 time before food access (food anticipatory activity; FAA), and quantification of activity and body temperature, before food access (n = 4). Serum free fatty acid levels at ZT22 and ZT3 in wild type and coconut oil infused *L Per2*$^{-/-}$ mice. All values are mean ± SEM. One-way ANOVA with Tukey's post test. *$p < 0.05$. (**d**) Western blot showing PER2 levels in the liver after application of NaCl, βOHB, and Sodium pyruvate respectively. GAPDH was used as control for each genotype and treatment.

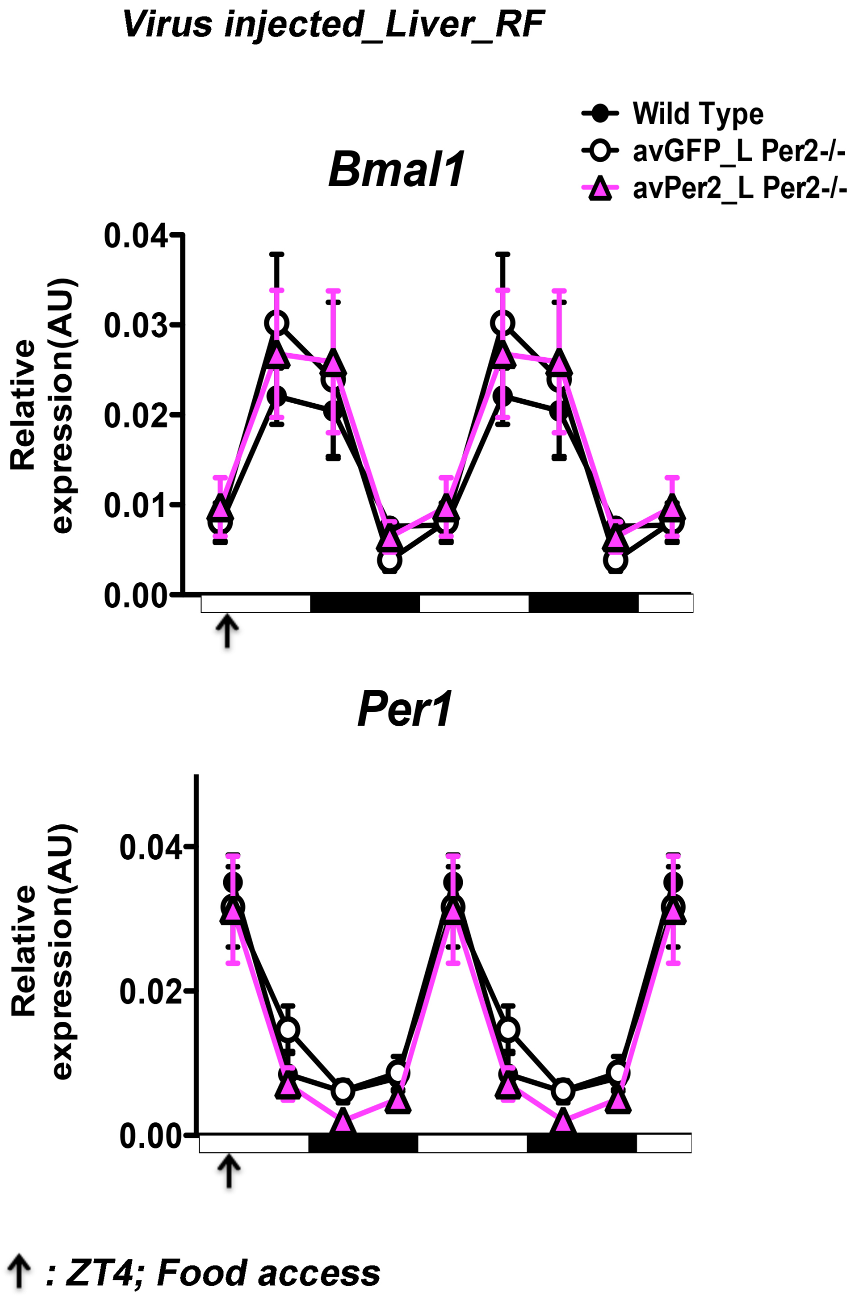

Supplementary Figure 6

Supplementary Figure 6 Overexpression of Per2 in the liver does not alter *Bmal1* and *Per1* expression. Double-plotted (Mean ± SEM, n = 3-4) mRNA levels of circadian oscillator components *Bmal1 and Per1* in liver of wild-type, avGFP_*L Per2*$^{-/-}$ *and* avPer2_*L Per2*$^{-/-}$ mice under restricted feeding conditions at different times of the day. Transcript levels were measured by quantitative PCR and normalized to *Gapdh*. Arrows indicate the time when food access started.

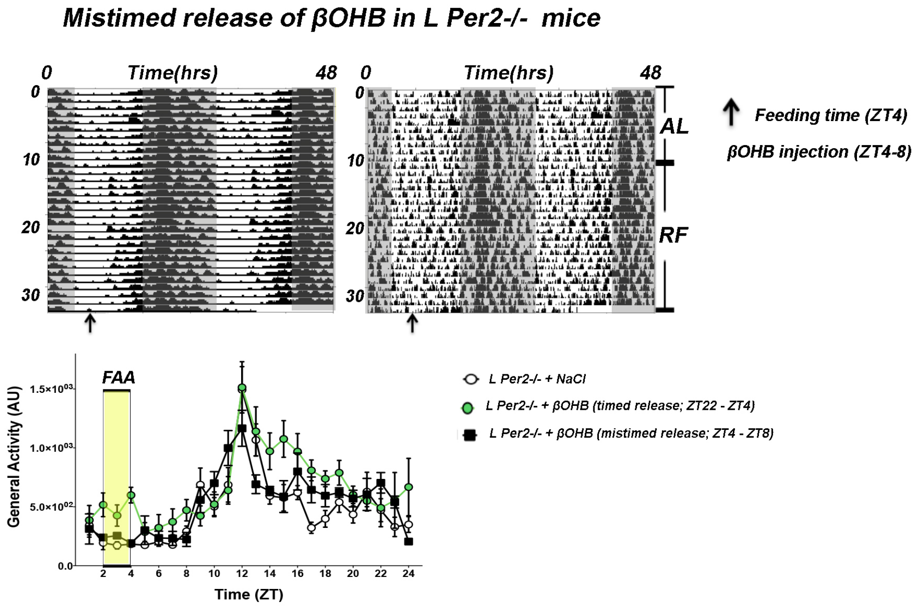

Supplementary Figure 7

Supplementary Figure 7 Mistimed βOHB release does not restore FAA in *L Per2*$^{-/-}$ mice. Top: Example of double plotted activity and body temperature actograms of mistimed release of βOHB (ZT4-ZT8) in *L Per2*$^{-/-}$ mice. Arrows indicate the start of food access. Bottom: quantification of the activity profile (Mean ± SEM, n = 4-6), yellow rectangle represents ZT2-4 time before food access (food anticipatory activity; FAA).

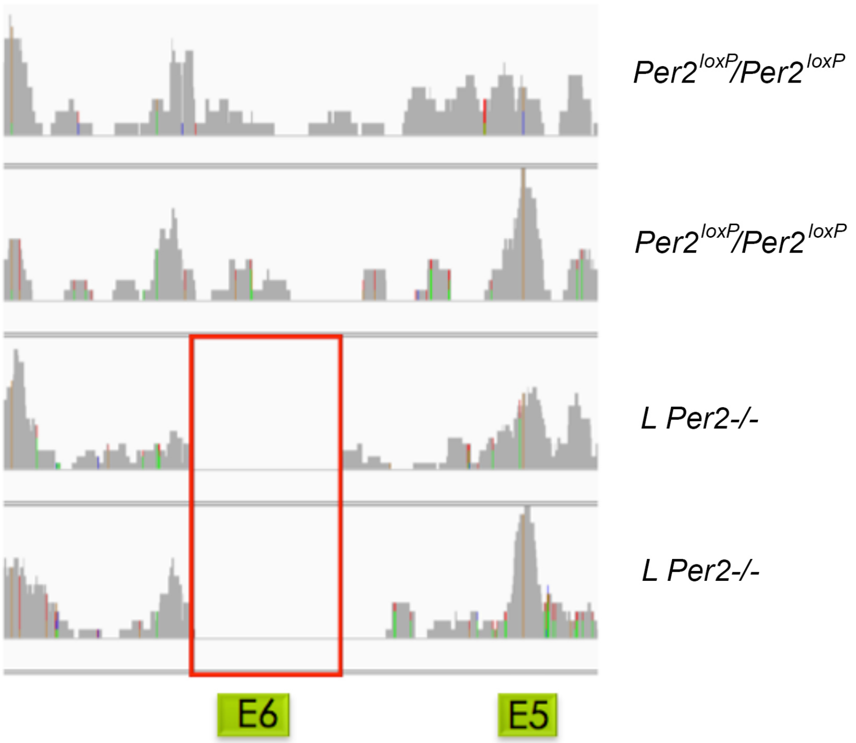

Supplementary Figure 8

Supplementary Figure 8 Liver-specific deletion of exon 6 from *Per2*. Deletion was obtained by crossing *Per2*^{fl}/*Per2*^{fl} and liverCre^{+} (*Alb1-cre*) mice and was verified by Illumina ultra-deep sequencing. Read height was adjusted to 25 for all four tracks. A red box indicates deletion of exon 6.

a

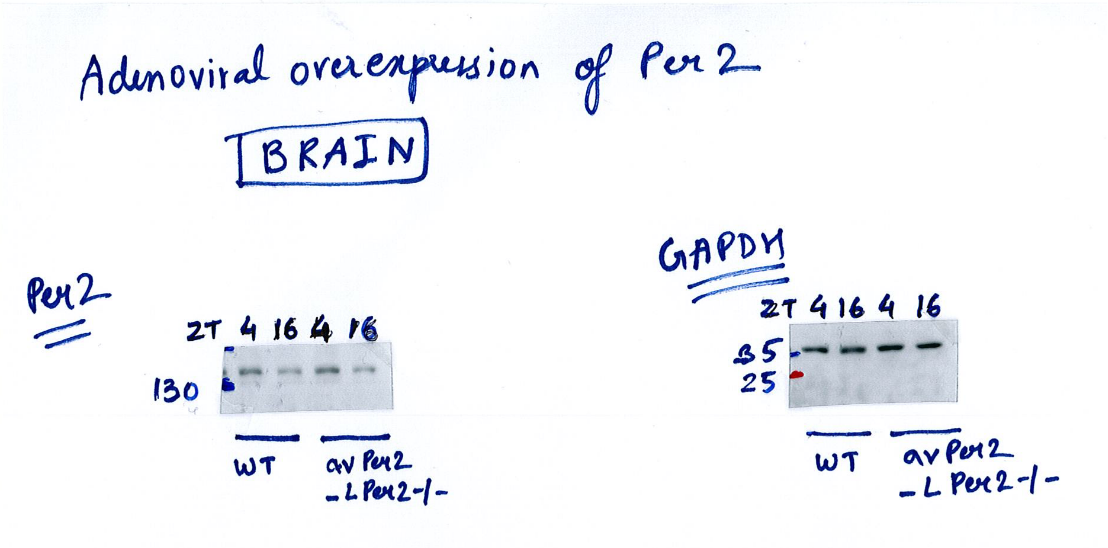
Adenoviral overexpression of Per2
BRAIN
Per2
ZT 4 16 4 16
130
WT
av Per2
- L Per2-/-
GAPDH
ZT 4 16 4 16
35
25
WT
av Per2
- L Per2-/-

b

Adenoviral overexpression of Per2

LIVER

Per2

LPer2-/-

ZT 4 10 16 22

250

130

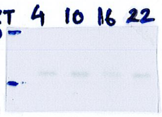

~~avPer2-/-~~

avPer2_LPer2-/-

250

130

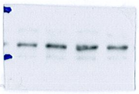

avGFP_L Per2-/-

130

100

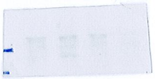

Wildtype

130

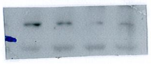

GFP

35

25

15

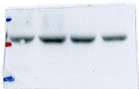

GAPDH LPer2-/-

ZT 4 10 16 22

35

25

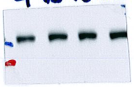

avPer2_LPer2-/-

55

35

25

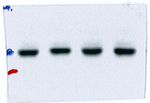

avGFP_LPer2-/-

35

25

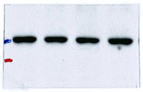

wildtype

35

25

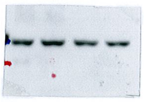

~~BRAIN~~

~~wildtype~~

~~wildtype~~

~~avPer2_LPer2-/-~~

~~avPer2_LPer2-/-~~

c

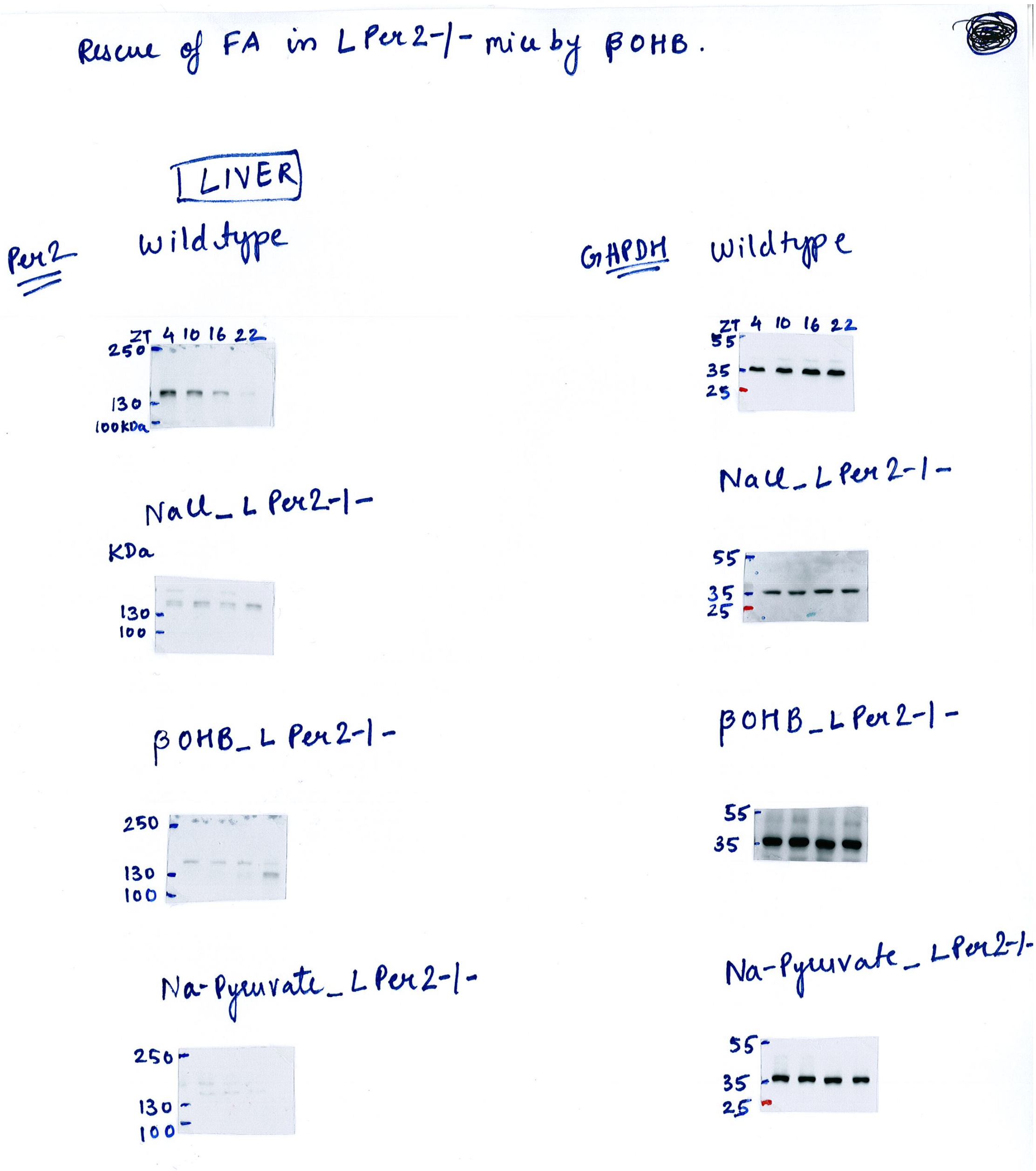

Rescue of FA in L Per2-/- mice by βOHB.
LIVER
Per2
wild type
ZT 4 10 16 22
250
130
100kDa
Nacl_L Per2-/-
KDa
130
100
βOHB_L Per2-/-
250
130
100
Na-Pyruvate_L Per2-/-
250
130
100
GAPDH
wildtype
ZT 4 10 16 22
55
35
25
Nacl_L Per2-/-
55
35
25
βOHB_L Per2-/-
55
35
Na-Pyruvate_L Per2-/-
55
35
25

d

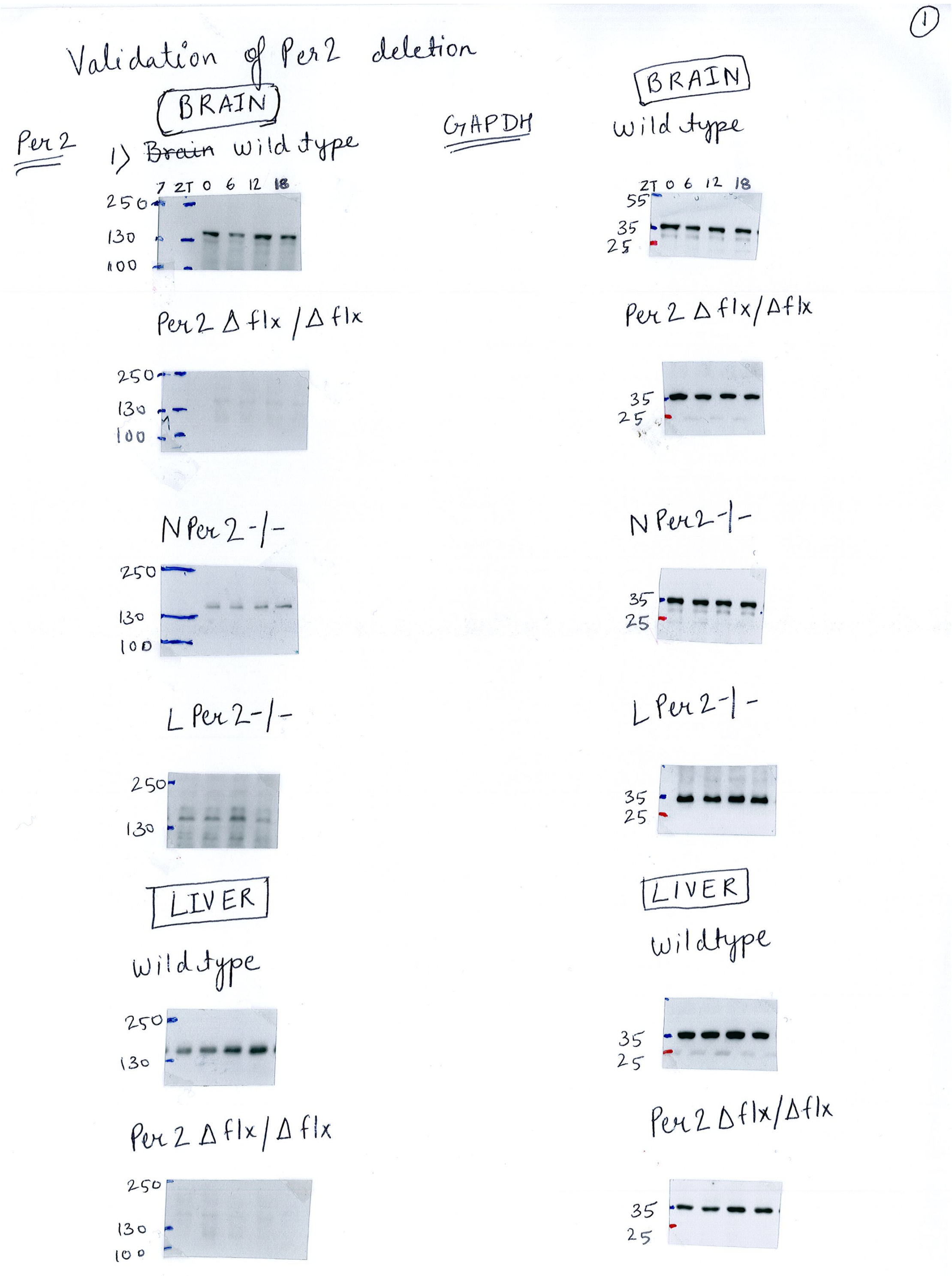
1
Validation of Per2 deletion
Per2
BRAIN
1) ~~Brain~~ wild type
ZT 0 6 12 18
250
130
100
GAPDH
BRAIN
wild type
ZT 0 6 12 18
55
35
25
Per2 Δflx/Δflx
250
130
100
Per2 Δflx/Δflx
35
25
N Per2-/-
250
130
100
N Per2-/-
35
25
L Per2-/-
250
130
L Per2-/-
35
25
LIVER
wild type
250
130
LIVER
wildtype
35
25
Per2 Δflx/Δflx
250
130
100
Per2 Δflx/Δflx
35
25

e

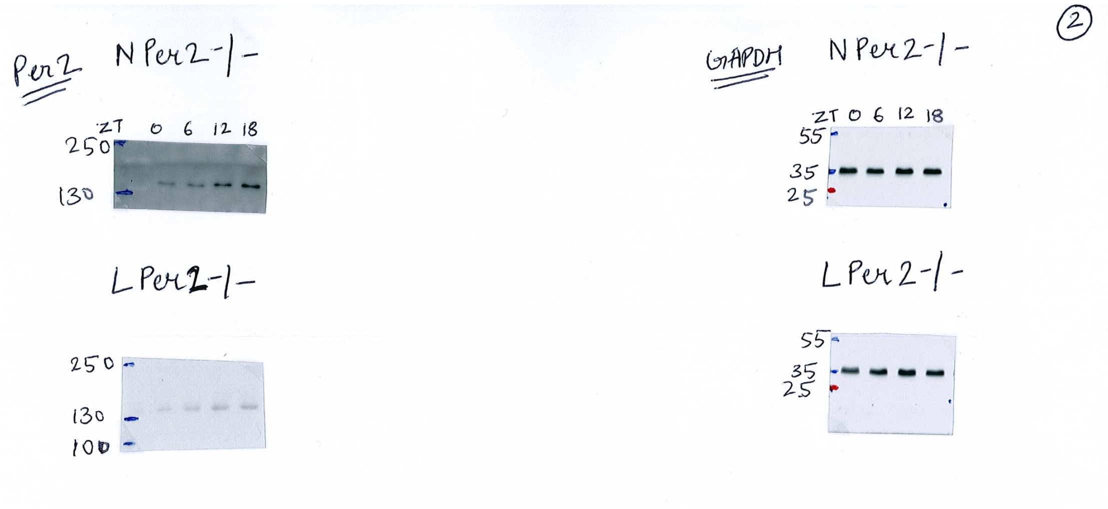

Supplementary Figure 9 Original blots corresponding to Fig. 2a and Supplementary Figure 3a (a-b), corresponding to Fig. 4b and Supplementary Figure 5d (c), and corresponding to Fig. 1c and Supplementary Figure 1a (d-e).

Supplementary Table 1

Sample ID	Number of sequences (fastq file)	Percentage of mapped sequences
WN1_S1_L001-2-3-4	19010627	85.06%
WN2_S2_L001-2-3-4	49333262	81.51%
WN3_S3_L001-2-3-4	47368922	80.25%
WR1_S4_L001-2-3-4	16265286	80.23%
WR2_S5_L001-2-3-4	42256984	82.35%
WR3_S6_L001-2-3-4	50222525	80.87%
PN1_S7_L001-2-3-4	26186826	81.48%
PN2_S8_L001-2-3-4	23336101	80.50%
PN3_S9_L001-2-3-4	49361533	81.10%
PR1_S10_L001-2-3-4	19992237	81.34%
PR2_S11_L001-2-3-4	55615597	80.38%
PR3_S12_L001-2-3-4	45873256	78.46%

The table shows the detailed characterization of the files. All files have been analyzed with FastQC (Andrews S. 2012) in order to demonstrate the quality of the data.

Supplementary Table 2

Gene	Sense primer	Anti-sense primer	TM probe (5' FAM: BHQ1 3')
Gapdh	CATGGCCTTCCGTGTTCCTA	CCTGCTTCACCACCTTCTTGA	CCGCCTGGAGAAACCTGCCAAGTAA
Per2	TCCACAGCTACACCACCCCTTA	TTTCTCCTCCATGCACTCCTGA	CCGCTGCACACACTCCAGGGCG
Hmgcs2	TGCCCTGGTGGTCTGTGGTG	GCCTTGGGCCCGATCAGCAT	CCCGAGTGGTAACGCCCGCCCC
Cpt1a	CCAGGCTACAGTGGGACA	GCCAAAGGTGTCAAATGGG	CATCCCTAAGCAGTGCCAGTT
Bmal1	CCAAGAAAGTATGGACACAGACAAA	GCATTCTTGATCCTTCCTTGGT	TGACCCTCATGGAAGGTTAGAATATGCAGAA
Clock	TTGCTCCACGGGAATCCTT	GGAGGGAAAGTGCTCTGTTGTAG	ACACAGCTCATCCTCTCTGCTGCCTTTC
Per1	GGCATGATGCTGCTGACCACG	GGTGGGGATGGGCTCTGTGA	TGGCCCTCCCTCACCTTAGCCTGTTCCT
RORα	CCTTCACCAACGGAGAGACT	CCAGGT GGGATTTGGATATG	TCCCCAACCGTGTCCATGGC
Rev-erbα	GAAGTGTCTCTCCGTTGGCATGTCT	CGCTCTGCATCTCGGCAAGCAT	CTGTGCGTTTTGGGCGCATCCCCAAG
Cry1	CTGGCGTGGAAGTCATCGT	CTGTCCGCCGAGTTCTATG	CGCATTTCACATACACTGTATGACCTGGACA

The table enlists the primers and TaqMan probes used for quantitative PCR analysis.
